# PaIRKAT: A pathway integrated regression-based kernel association test with applications to metabolomics and COPD phenotypes

**DOI:** 10.1371/journal.pcbi.1008986

**Published:** 2021-10-22

**Authors:** Charlie M. Carpenter, Weiming Zhang, Lucas Gillenwater, Cameron Severn, Tusharkanti Ghosh, Russell Bowler, Katerina Kechris, Debashis Ghosh

**Affiliations:** 1 Department of Biostatistics and Informatics, University of Colorado Denver, Anschutz Medical campus, Denver, Colorado, United States of America; 2 Syneos Health, Morrisville, North Carolina, United States of America; 3 Computational Bioscience Program, University of Colorado Denver, Anschutz medical campus, Denver, Colorado, United States of America; 4 Department of Medicine, National Jewish Health, Denver; University of Colorado Denver, Anschutz Medical Campus, Denver, Colorado, United States of America; Heidelberg University, GERMANY

## Abstract

High-throughput data such as metabolomics, genomics, transcriptomics, and proteomics have become familiar data types within the “-omics” family. For this work, we focus on subsets that interact with one another and represent these “pathways” as graphs. Observed pathways often have disjoint components, i.e., nodes or sets of nodes (metabolites, etc.) not connected to any other within the pathway, which notably lessens testing power. In this paper we propose the Pathway Integrated Regression-based Kernel Association Test (PaIRKAT), a new kernel machine regression method for incorporating known pathway information into the semi-parametric kernel regression framework. This work extends previous kernel machine approaches. This paper also contributes an application of a graph kernel regularization method for overcoming disconnected pathways. By incorporating a regularized or “smoothed” graph into a score test, PaIRKAT can provide more powerful tests for associations between biological pathways and phenotypes of interest and will be helpful in identifying novel pathways for targeted clinical research. We evaluate this method through several simulation studies and an application to real metabolomics data from the COPDGene study. Our simulation studies illustrate the robustness of this method to incorrect and incomplete pathway knowledge, and the real data analysis shows meaningful improvements of testing power in pathways. PaIRKAT was developed for application to metabolomic pathway data, but the techniques are easily generalizable to other data sources with a graph-like structure.

## Author summary

PaIRKAT is a tool for improving testing power on high dimensional data by including graph topography in the kernel machine regression setting. Studies on high-dimensional data can struggle to include the complex relationships between variables. The semi-parametric kernel machine regression model is a powerful tool for capturing these types of relationships. They provide a framework for testing for relationships between outcomes of interest and high dimensional data such as metabolomic, genomic, or proteomic pathways. Our paper proposes a kernel machine method for including known biological connections between high dimensional variables by representing them as edges of ‘graphs’ or ‘networks.’ It is common for nodes (e.g., metabolites) to be disconnected from all others within the graph, which leads to meaningful decreases in testing power when graph information is ignored. We include a graph regularization or ‘smoothing’ approach for managing this issue. We demonstrate the benefits of this approach through simulation studies and an application to the metabolomic data from the COPDGene study.

This is a *PLOS Computational Biology* Methods paper.

## Introduction

Metabolomics is the study of the metabolite composition of a cell, tissue, or biological fluid. Leading metabolomic experimental techniques such as liquid or gas chromatography coupled with mass spectrometry (LC-MS or GC-MS) and nuclear magnetic resonance (NMR) spectroscopy can capture the abundance of all metabolites within a cell (the metabolome). These technologies provide high-throughput data similar to other familiar -omics datatypes such as genomics, transcriptomics, and proteomics. An important advantage of metabolomics over other -omics data is its proximity to biological phenotypes[1]. While genomic or proteomic data are vital pieces for understanding the progression from DNA to phenotype, the metabolites are the end products of the enzymatic reactions of a cell[2]. The metabolome is comprised of exogenous (environmentally derived) and endogenous (genetically regulated) metabolites which can be used as biomarkers for the current phenotypic state of a cell or organism.

Like other -omics data, careful considerations of the metabolome’s unique characteristics are required to fully leverage it for biological insights. Specifically, metabolites are known to be related directly and indirectly by enzymatic reactions within a metabolomic pathway. Clustering methods have been developed to incorporate this connectivity into the primary analysis to avoid this two-step approach. These include Bayesian methods for metabolite clustering based on peak detection[3,4] and *ad hoc* methods based on singleton metabolite presence[5]. For this work, we choose to group subsets of metabolites that interact with one another and represent these pathways as *graphs* or *networks*. Throughout this paper we will use the term *graph* and *network* interchangeably. Open source databases with metabolomic pathway documentation such as the Kyoto Encyclopedia of Genes and Genomes (KEGG), the Human Metabolome Database (HMDB), Reactome, OmniPath, and WikiPathways are growing resources[6–10], and the pathways within these databases are easily translated to graphs to be used in downstream analyses.

The semiparametric kernel machine regression method[11,12] has gained popularity in many areas of biomedical research such as genomics, microbiome analysis, and neuroimaging[13–15]. One reason for its popularity is that it provides a computationally scalable method of classification and regression through the introduction of a *kernel* function. Another is that it provides a setting for formal statistical estimation and testing procedures for high-dimensional data sources, often using a score statistic. Formal statistical tests are useful for metabolomic research, as a goal is often identifying specific metabolites and pathways for further inquiry. At a high level, kernel machines test for relationships between an outcome and a set of predictors by testing if variation between the two correspond with one another.

A hurdle more unique to metabolomics is the high levels of sparsity in individual metabolites and pathway connectivity. While metabolomic databases (e.g., KEGG, HMDB) are growing, none are considered complete. Data generating techniques like LC-MS and GC-MS are also imperfect technologies that may miss metabolite abundances that are too low[16]. Thus, pathway representations of metabolomic data are often sparse and disconnected, i.e., nodes or sets of nodes are not connected to any other within the pathway.

Disjoint nodes are of concern for graph-structured data. Techniques that force graphs to be fully connected by making small, uniform changes to the structure have been suggested for handling this issue[17,18]. However, it is understood that these alterations impose new challenges by changing the subspaces spanned by the graph. Works by Schaid [19] as well as Freytag et al. [20] developed a network-based kernel where similarity is defined directly from the network structure. These methods and others like it are tailored to genome-wide association studies and not applicable to other omics data. Freytag also imposes “as much noise as necessary” within the network to ensure positive semidefinite matrices which is something we aim to avoid. In fact, our proposal dampens out noisy features of the graph. The *PIMKL* method works with pathways within the metabolome by combining them through a weighted summed kernel[21]. These weights provide insight into the importance of each sub-pathway, but this does not surmount to the level of evidence gathered from a direct comparison between specific pathways and phenotype.

In this paper we propose the Pathway Integrated Regression-based Kernel Association Test (PaIRKAT), a new kernel machine regression method for incorporating known pathway information into the semi-parametric kernel regression framework. In addition, PaIRKAT contributes an application of a graph kernel regularization method for overcoming sparse connectivity and disjoint pathways. To our knowledge, this is the first method to incorporate graph regularization into a kernel regression test. PaIRKAT allows for tests of association with phenotypes and the specific pathways while integrating pathway structure, and, instead of adding small amounts of noise, this approach dampens noisy components of a pathway while preserving biologically relevant signals. This leads to improved testing power and better overall biomarker detection. We evaluate these methods through several simulation studies and an application to real metabolomics data from the COPDGene[22] study.

## Results

### Method overview

Here we provide the main steps of PaIRKAT and provide an overview of the ideas behind them. The method is described in full in **Methods and Models**. The primary goal of PaIRKAT is to include the topographical information of graph structured data into the kernel machine regression model. We use the semiparametric kernel machine model[11,12,23] to test for relationships between the phenotype of interest, ***Y***, and a high dimensional set, ***Z***, while controlling for important covariates, ***X***, in the model *g*(***Y***) = **Xβ**+*h*(***Z***)+***ϵ***. In this model *h*(·) is a positive semidefinite kernel function that transforms ***Z*** to an appropriate feature space.

Omics data (metabolomics, genomics, etc.) can often be represented as a graph with edges representing biological interactions between the nodes (metabolites, etc.). Freytag et al. and Schaid both define a kernel directly from the graph structure where higher proximity within the pathway gives a higher similarity score [19,20]. This has been coined a ‘guilt by association’ approach [24] and has been proven effective empirically. These methods use a map from SNPs to genes to formulate similarity matrices, making them unapplicable to other types of studies. PaIRKAT also uses the ‘guilt by association’ paradigm but relies on a graph’s *regularized normalized Laplacian* as the measure of proximity within the pathway. Then any appropriate kernel can be applied for testing making it more generally applicable than other similar approaches.

We explored the utility of incorporating the Laplacian directly into the kernel machine but found it to be ineffective using simulation studies. Instead, we transform $\tilde{L}$ using methods designed to dampen noisy aspects of a graph while preserving its biologically relevant features[25,26]. The PaIRKAT method is to include this *regularized normalized Laplacian*, ${\tilde{L}}_{R}$, in the model through the kernel function as $g(Y)=X\beta +h(Z{\tilde{L}}_{R})+\epsilon$. Tests for relationships between ***Y*** and $h(Z{\tilde{L}}_{R})$ are performed using an adjusted score statistic[23] and Davies’ method for estimating distributions of linear combinations of *χ*^2^ variables[27].

### Simulation results

A complete description of our simulation study can be found in **Methods and Models**, but we give a brief synopsis of the simulation scheme. We first randomly generated a graph. Second, we randomly generated features, ***Z***, from multivariate normal distribution with a covariance structure derived from the graph. Lastly, we randomly generated a normally distributed outcome, ***Y***, with a mean based on a linear relationship between the columns of ***Z***. We performed tests ignoring graph topography, including graph topography in the kernel function via the normalized Laplacian ($\tilde{L}$), and our proposed method PaIRKAT of including graph topography in the kernel function via the regularized Laplacian (${\tilde{L}}_{R}$). Our simulations aimed to assess how sensitive our method is to incomplete and/or incorrect graph information. We also compare the power of our method to two simple competing approaches: an F-test on all principal components (PCs) of ***Z*** [28] and the minimum Simes’ adjusted p-value[29] from univariate tests on ***Z*** (Univariate Simes).

Type I error rates for PaIRKAT are summarized in Tables 1, 2, 3 and 4. The type I error rates for tests using a graph’s normalized Laplacian, $\tilde{L}$ (see **Methods and Models** section for definition), are summarized in S1, S2, S3, and S4 Tables. The type I error rate of ≈0.05 is maintained throughout all simulation scenarios.

**Table 1:** Type 1 error rates using all pathway information, i.e., no nodes or edges were dropped for these simulations. “*Perfect*” indicates calculating ${\tilde{L}}_{R}$ from the graph used to generate the data. “*Mismatch*” indicates the percentage of direct edges that were incorrect. Error rates were calculated from score tests on 1000 simulated data sets. All simulations used graphs with 15, 30, or 45 nodes. “*Complete Mismatch*” indicates 100% mismatch.

	Pathway size		
	15	30	45
*Perfect*	0.0482	0.0529	0.0568
*10% Mismatch*	0.0498	0.0494	0.0474
*40% Mismatch*	0.0487	0.0525	0.0464
*70% Mismatch*	0.0502	0.0512	0.0511
*Complete Mismatch*	0.0487	0.0511	0.0494
*No Pathway*	0.0580	0.0540	0.0530
*Principal Component*	0.0484	0.0543	0.0558
*Univariate Simes*	0.0490	0.0513	0.0507

**Table 2:** Type 1 error rates using pathways with 5% missing edges. Error rates were calculated from score tests on 1000 simulated data sets using graphs with 15, 30, or 45 nodes. The graph used to simulate ***Z*** and ***Y*** was of medium edge density, while the graph used to test was of low density. The low-density graphs are drawn from the Barabasi-Albert model with edge density 0.13, 0.07, and 0.04 for graphs with 15, 30, and 45 nodes, respectively. Medium edge density graphs are created by giving any 2 nodes without a direct edge between them a 5% chance of becoming directly connected. This creates graphs with an average edge density of 0.18, 0.12, and 0.09 for graphs with 15, 30, and 45 nodes, respectively. “*Perfect*” indicates calculating ${\tilde{L}}_{R}$ from the graph without changing remaining edges. “*Mismatch*” indicates the percentage of remaining direct edges that were incorrect. “*Complete Mismatch*” indicates 100% mismatch.

	Pathway size		
	15	30	45
*Perfect Network*	0.0497	0.0491	0.0463
*10% Mismatch*	0.0478	0.0479	0.0485
*40% Mismatch*	0.0465	0.0510	0.0536
*70% Mismatch*	0.0518	0.0523	0.0486
*Complete Mismatch*	0.0491	0.0539	0.0463
*No Network*	0.0480	0.0440	0.0390
*Principal Component*	0.0507	0.0489	0.0494
*Univariate Simes*	0.0515	0.0494	0.0477

**Table 3:** Type 1 error rates using pathways with 15% missing edges. Error rates were calculated from score tests on 1000 simulated data sets using graphs with 15, 30, or 45 nodes. The graph used to simulate ***Z*** and ***Y*** was of high edge density, while the graph used to test was of low density. The low-density graphs are drawn from the Barabasi-Albert model with edge density 0.13, 0.07, and 0.04 for graphs with 15, 30, and 45 nodes, respectively. High edge density graphs are created by giving any 2 nodes without a direct edge between them a 15% chance of becoming directly connected. This creates graphs with an average edge density of 0.26, 0.21, and 0.19 for graphs with 15, 30, and 45 nodes, respectively. “*Perfect*” indicates calculating ${\tilde{L}}_{R}$ from the graph without changing remaining edges. “*Mismatch*” indicates the percentage of remaining direct edges that were incorrect. “*Complete Mismatch*” indicates 100% mismatch.

	Pathway size		
	15	30	45
*Perfect Network*	0.0508	0.0538	0.0456
*10% Mismatch*	0.0541	0.0519	0.0521
*40% Mismatch*	0.0495	0.0486	0.0478
*70% Mismatch*	0.0514	0.0506	0.0524
*Complete Mismatch*	0.0504	0.0523	0.0490
*No Network*	0.0430	0.0530	0.0510
*Principal Component*	0.0525	0.0509	0.0481
*Univariate Simes*	0.0499	0.0491	0.0459

**Table 4:** Type 1 error rates using pathways with dropped nodes. Error rates were calculated from score tests on 1000 simulated data sets using graphs 15, 30, or 45 nodes initially. The graph used to simulate ***Z*** and ***Y*** contained all nodes. Nodes with degree below the 25^th^ percentile within a graph had a 25% chance of being dropped before testing. “*Perfect*” indicates calculating ${\tilde{L}}_{R}$ from the graph without changing edges between remaining nodes. “*Mismatch*” indicates the percentage of direct edges between remaining nodes that were incorrect. “*Complete Mismatch*” indicates 100% mismatch.

	Pathway size		
	15	30	45
*Perfect Network*	0.0480	0.0513	0.0494
*10% Mismatch*	0.0499	0.0489	0.0476
*40% Mismatch*	0.0492	0.0495	0.0501
*70% Mismatch*	0.0522	0.0511	0.0500
*Complete Mismatch*	0.0481	0.0488	0.0483
*No Network*	0.0420	0.0490	0.0530
*Principal Component*	0.0505	0.0476	0.0501
*Univariate Simes*	0.0481	0.0502	0.0502

**Fig 1:**
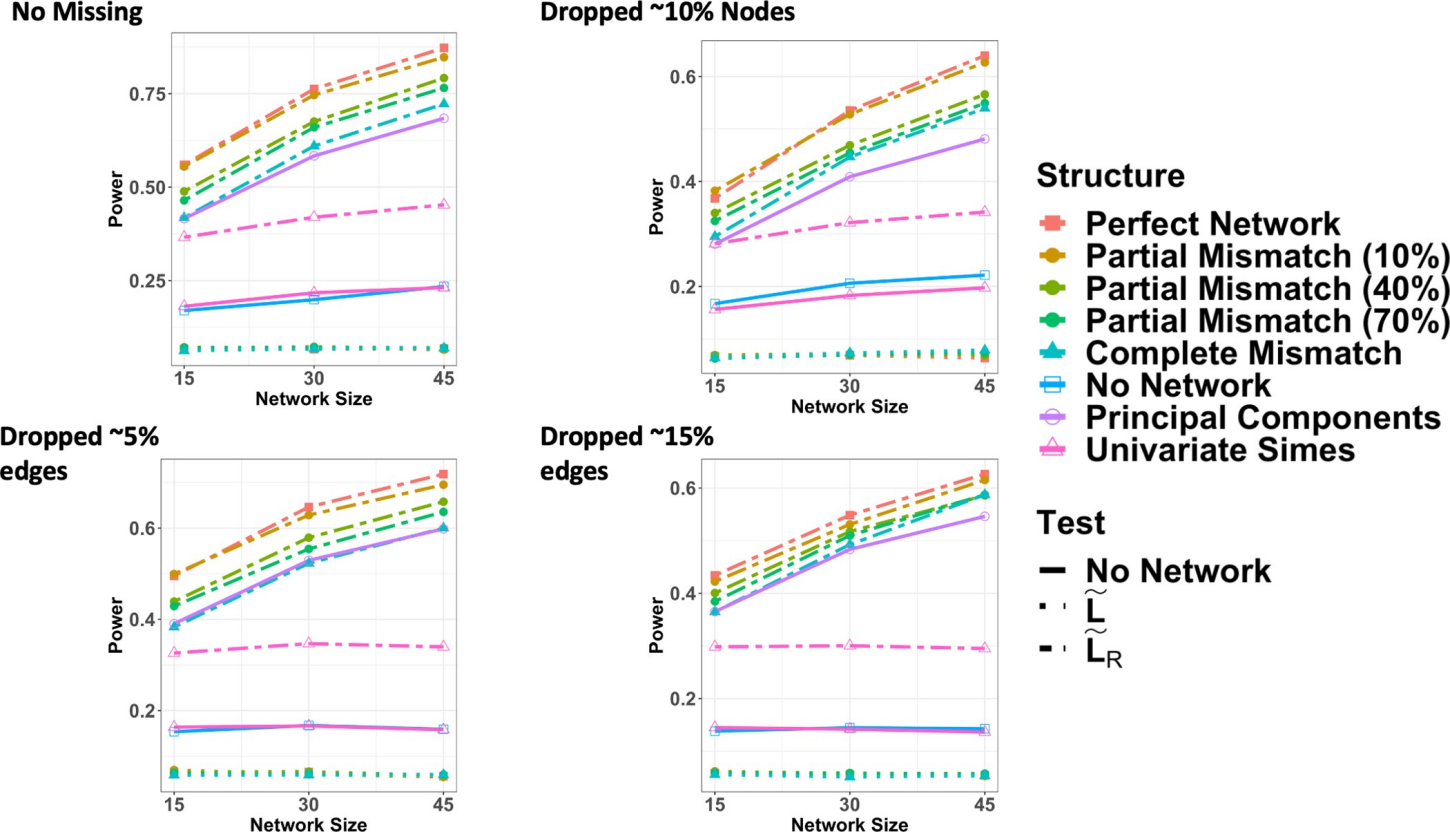
Power curves from the four pathway *knowledge* and 6 pathway *structure* simulation scenarios. Power curves were all calculated from score tests on 1000 simulated data sets using graphs with 15, 30, or 45 nodes. Power curves assuming complete pathway knowledge with no dropped edges or nodes are displayed in a). For (b) and (c), the graph used to simulate ***Z*** and ***Y*** was of medium or high density, respectively, while the graph used to test was of low density. Medium and high edge density graphs used for data generation had ~5% and ~15% more edges, respectively, than the low-density graph used for testing. The power curve generated assuming missing nodes (d) used all graph nodes to generate ***Z*** and ***Y***. Then nodes (and corresponding columns of ***Z***) with degree below the 25^th^ percentile within a graph had a 25% chance of being dropped before testing.

**Fig 2:**
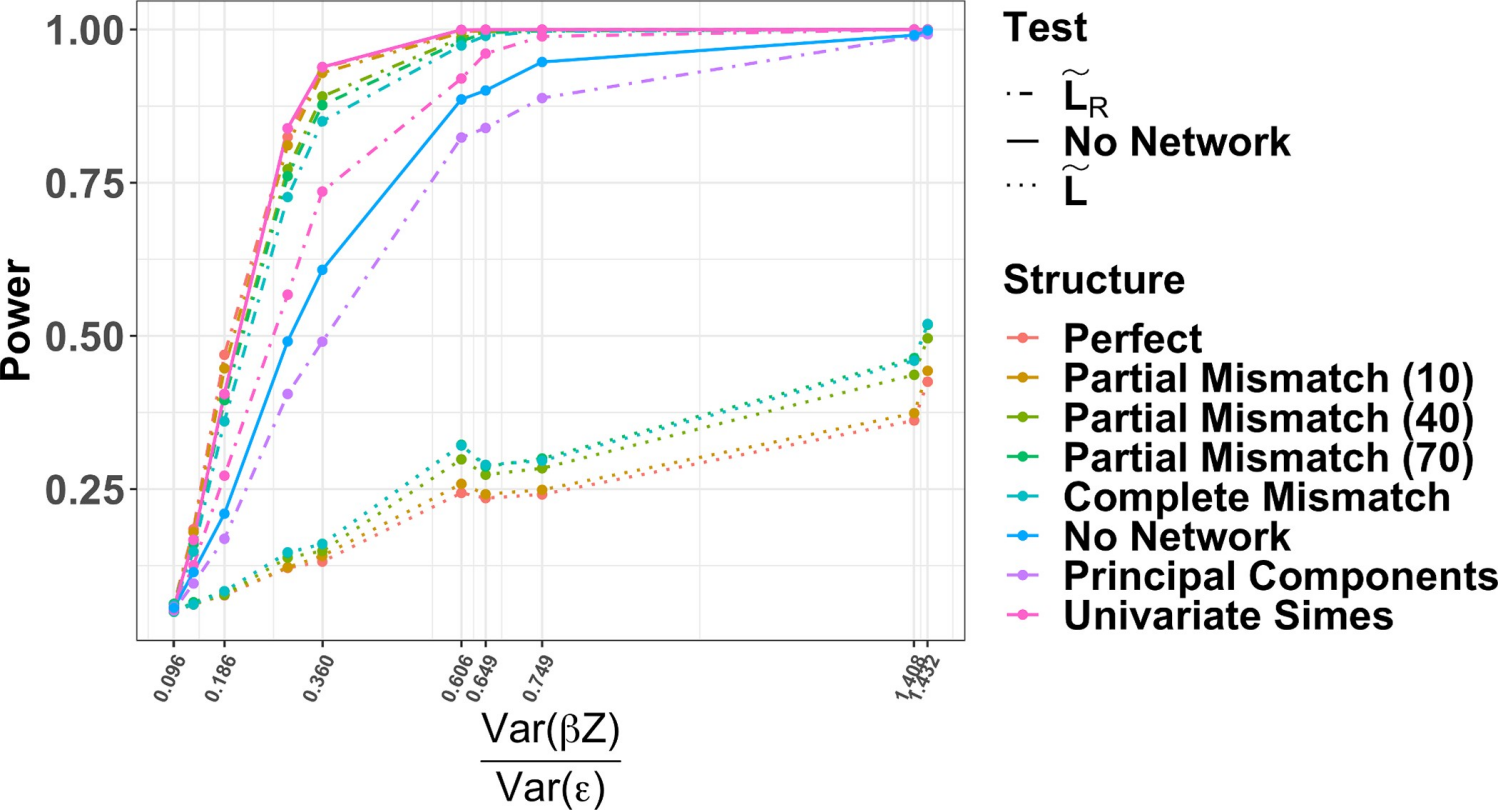
Signal to Noise Ratio. Power curves from increasing the signal to noise ratio while assuming complete pathway knowledge. The signal to noise ratio was calculated as the as the ratio between the overall variance in ***Y***, $Var(\beta_{0}+\sum_{j=1}^{p}\beta_{j}Z_{ij})$, and the overall residual variance, $Var[Y_{i}-(\beta_{0}+\sum_{j=1}^{p}\beta_{j}Z_{ij})]$. Each power calculation comes from score tests on 1000 simulated data sets using graphs with 30 nodes.

The power curves for all pathway structures and competing methods while simulating complete knowledge, missing edges, and missing nodes are displayed in Fig 1. Having a perfect pathway structure provides the most power. Relationships between an outcome and pathway are easier to detect in larger pathways. The more incorrect direct edges in the pathway, the lower the overall power. The univariate Simes was improved by including ${\tilde{L}}_{R}$. Using the PCs of ***Z*** and $Z{\tilde{L}}_{R}$ gave the exact same power, which is expected from a basis transformation, and performed similarly to a completely incorrect edge structure. Clearly, any correct information from the graph improved power overall. We also see that increasing the overall signal to noise ratio improves power for all pathway structures (Fig 2). PaIRKAT (${\tilde{L}}_{R}$) achieves approximately 80% power at a signal to noise ratio around 0.32, whereas ignoring network information requires a signal to noise ratio over twice that, about 0.70 and only including the Laplacian never achieves 80% power (Fig 2). The univariate Simes’ test performed as well as PaIRKAT with perfect pathway knowledge. This is unsurprising since all ***z***_*i*_ are related to the outcome in our simulations.

### COPDGene analysis results

A complete description of these analyses can be found in **Methods and Models**, but here we give a brief description of the outcome variables we analyzed. We create models for two phenotypes from the COPDGene study[22]: (1) percent emphysema and (2) the ratio of post-bronchodilator forced expiratory volume at one second divided by forced vital capacity (FEV_1_/FVC). To normalize FEV_1_/FVC, we use the following log ratio transformation, $log\left(\frac{FEV_{1}/FVC}{1-FEV_{1}/FVC}\right)$. This is referred to as the “log FEV_1_/FVC ratio” for simplicity. We test for associations between 28 pathways and each outcome under the same three conditions in the simulation study: ignoring graph topography, including graph topography via the normalized Laplacian ($\tilde{L}$), and our proposed method PaIRKAT of including graph topography via the regularized Laplacian (${\tilde{L}}_{R}$).

Including the metabolites’ regularized graphs had large impacts on the associations between the log FEV1/FVC ratio and several subsets of metabolites. For the 28 pathways tested, power was improved for 17 pathways when using PaIRKAT vs. using $\tilde{L}$ or ignoring pathway information. Of note, the strength of the associations between the log FEV1/FVC ratio and the *ABC transporters*, the *arginine and proline metabolism*, the *cysteine and methionine metabolism*, the *pyrimidine metabolism*, the *glycine*, *serine*, *and threonine metabolism*, and the *neuroactive ligand-receptor interaction* metabolite subsets increased dramatically. The average p-value was also lower for 12 pathways with using $\tilde{L}$ vs. ignoring pathway information. S1 Fig displays the p-values from the kernel regression tests for associations between the log FEV1/FVC and the 28 pathways of interest for each subsample size.

Including the metabolites’ regularized graphs also had impacts on the associations between percent emphysema and several subsets of metabolites. For the 28 subsets of metabolites tested, power was improved for 17 pathways when using PaIRKAT vs. including $\tilde{L}$ or ignoring pathway information. Of note, the strength of the associations between percent emphysema and the *ABC transporters*, the *β-alanine metabolism*, the *neuroactive ligand-receptor interaction*, the *glycine*, *serine and threonine metabolism*, and the *histidine metabolism* metabolite subsets increased dramatically when using PaIRKAT vs. ignoring pathway information. The average p-value was also lower for the same 5 pathways with using $\tilde{L}$ vs. ignoring pathway information. However, there was still not a significant result from any method for 4 of these pathways, and PaIRKAT provided similar power for the fifth. S2 Fig displays the p-values from the kernel regression tests for associations between percent emphysema and the 28 pathways of interest for each subsample size.

**Fig 3:**
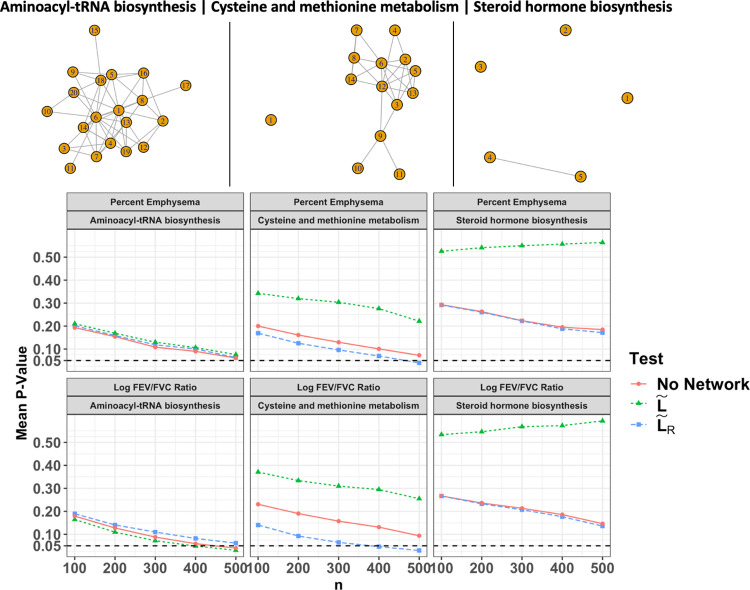
Selected results from COPDGene subset analysis. Average p-values from kernel regressing tests that do not include pathway information (No Laplacian, red circles), include pathway information through a normalized Laplacian ($\tilde{L}$, green triangles), and include pathway information through a regularized normalized Laplacian (${\tilde{L}}_{R}={\left(I+\tau \tilde{L}\right)}^{-1}$, blue squares) are displayed. P-values were averaged over 100 random subsets of size 100, 200, 300, 400, and 500 from the COPDGene dataset. *τ* was set to 1 for all tests that used ${\tilde{L}}_{R}$. The 3 pathways selected illustrate the expected results in fully connected (left), partially disconnected (middle), and sparse (right) graphs.

Fig 3 displays results from 3 pathways selected to illustrate PaIRKAT’s impact on power for fully connected (left column), partially disconnected (middle column), and sparse (right column) graphs. For the *steroid hormone biosynthesis* pathway, an almost completely sparse pathway, we see virtually no differences between PaIRKAT and ignoring pathway connectivity. We also see relatively small differences between all three methods for the fully connected *aminoacyl-tRNA biosynthesis* pathway. The major impacts from PaIRKAT come when there are a few nodes or node subsets disjoint from the rest of the graph, as we see in the *cysteine and methionine metabolism*.

## Discussion

We have developed PaIRKAT, a method for incorporating pathway information under a kernel regression framework. Other methods to incorporate pathway connectivity via graph operations have been developed[20,21,26,30–32]. PaIRKAT enables the researcher to test on specified pathways instead of aggregating all pathways through a weighted kernel as in[21,30]. It can also handle disjointed pathways without adding in artificial noise to the network as in[17,18,20]. This allows the investigator to compile information from multiple sources, e.g., KEGG and HMDB. The regression framework also expands upon a method developed for classification[26]. It should be noted that the kernel framework is testing a global null, i.e., if any node covaries with the outcome the null hypothesis is rejected. See Goeman and Buhlmann[33] for a full discussion on whether or not this approach is appropriate for pathway based hypotheses.

Pathway misspecifications from incomplete data collection or imperfect canonical pathways within databases are common hurdles in -omics studies. We explored the sensitivity of the method by simulating data assuming incorrect pathway structures and incomplete pathway knowledge. These studies show that our method is highly robust to pathway misspecifications. In smaller pathways, we see that the partially mismatched structure with ~10% of direct edges being incorrect does as well as the perfect network structure. This is likely due to the very small change from the perfect structure in these cases, as a graph with only 15 nodes could easily be unchanged with only a 10% chance to change an edge. Furthermore, even with incorrect or incomplete pathway information, our method provides significantly improved power over ignoring pathway information while maintaining an appropriate type I error rate. We believe this is because many indirect connections between nodes are preserved, and these connections still provide more accurate information than incorrectly assuming independence among nodes.

One benefit of using PaIRKAT is improved power to identify pathways that are associated with clinical phenotypes. For example, an application to the COPDGene dataset using KEGG’s database of metabolic pathways also illustrated PaIRKAT’s ability to improve testing power over simply treating metabolites as independent (Figs 3, S1, and S2). The regularization technique was also able to handle pathways with few metabolites and/or disjoint components. Several tests had a notables boost in power from including pathway connectivity for both percent emphysema and the log FEV_1_/FVC ratio, and most pathways have been previously associated with COPD and lung function. Huang et al. linked environmental exposures, COPD risk, and metabolomic pathways, and found associations between COPD and the *histidine metabolism*, *cysteine and methionine metabolism*, and *β-alanine metabolism* pathways[34]. The *glycine*, *serine and threonine metabolism*, *aminoacyl-tRNA biosynthesis*, *pyrimidine metabolism*, *pantothenate and CoA biosynthesis*, pathways have all previously been associated with asthma[35]. The *β*-Alanine metabolism, ABC transporters, purine metabolism, pantothenate and CoA biosynthesis pathways were all differentially associated with COPD subclasses for patients with lung cancer[36]. Another study of the COPDGene dataset[22] using a two-step pathway enrichment approach found that the *purine metabolism*, *mineral absorption*, *arginine biosynthesis*, *aminoacyl-tRNA biosynthesis*, *ABC transporters* and *glycine*, *serine and threonine metabolism* pathways were all associated with various measures of lung function and increased COPD exacerbations[37]. The three *ABC transporters* have also been shown to be related to COPD in several murine knockout and human studies (see Chai et al.[38]). Finally, the *arginine biosynthesis* pathway has also been associated with COPD in multiple studies [39,40].

### Graph information

We used a non-proprietary version of KEGG available in R. The proprietary version of this database has more up to date information and could have resulted in different pathway structures for the COPDGene data set. There is also a substantial literature on data driven methods for deriving networks from omics data [41–47]. Chai et al. provide a nice review[48]. We leave the investigation of how these data-driven methods interact with ours to future research.

### Impacts of regularization

In simulation studies and real data analyses we saw meaningful improvements in power by including pathway information through a graph’s regularized normalized Laplacian, (PaIRKAT) when compared to ignoring the pathway information or using $\tilde{L}$. PaIRKAT was essential to maintaining testing power when graphs had disjoint nodes or sub-graphs. Using the normalized Laplacian, $\tilde{L}$, hindered testing performance compared to using PaIRKAT or ignoring the pathway information when a graph was disconnected. In connected graphs PaIRKAT, using $\tilde{L}$, and ignoring the pathway information all performed similarly in the real data analyses (Fig 3).

It is well established that $\tilde{L}$ is a symmetric and positive semidefinite matrix with eigen values 0≤*λ*_1_, *λ*_2_,…,*λ*_*p*_≤2, where the number of *λ*_*i*_ = 0 is the number of disjoint components of the undirected graph *G* (see **Methods and Models**). Therefore, graphs with very low connectivity, meaning many *λ*_*i*_ = 0, will not be as impacted by regularization since all *r*^−1^ (*λ*_*i*_ = 0) = *a* for some scalar *a*. In words, there is no extra information from a graph when most nodes are disconnected from one another (e.g., Fig 3, right column).

One limitation of this study is our focus on the Gaussian kernel. There has been success with other kernels for high dimensional data such as ones tailored to the data type [14,20] or simple linear and weighted linear kernels [23,49–51]. We have shown that including that including pathway information can improve the power of the Gaussian kernel and leave the impacts on other kernels to future work.

### Summary

In summary, our proposed method serves as a framework for including pathway information into a kernel machine regression test. We developed this method for application to metabolomic pathway data, but the techniques are easily generalizable to other data sources with a graph-like structure. It is important to examine the structure of a graph before applying a regularization step. Unique challenges arose from the sparsity present in many metabolomic pathways which can greatly hinder performance. We implement a graph regularization kernel to handle disconnected pathways. This regularization step is novel in the application of graph-based kernel machine regression to biological data. Our simulation studies illustrate the robustness of this method to improper and incomplete pathway knowledge. The method presented can provide powerful tests for associations between biological pathways and phenotypes of interest and will be helpful in identifying novel pathways for targeted clinical research.

## Methods and models

### The Kernel machine model

We assume that the data are properly filtered, imputed, and normalized for the methods described in this paper. Consider a dataset with observations from *n* subjects. Let ***Y*** be an *n*×1 vector representing a continuous or discrete phenotype of interest. Also let ***X*** be a *n*×*q* matrix of clinical covariates and ***Z*** be an *n*×*p* matrix of graph structure data. The phenotype can then be modeled through the following semiparametric model

g(Y)=Xβ+h(Z)+ϵ, 1)

where *g* is either the identity or *logit* link function, **β** is a *q*×1 vector of regression coefficients, ***ϵ*** is an *n*×1 vector of normally distributed error terms, and *h* is a kernel function. There are no parametric assumptions placed on *h* except that it lies in some feature space. This more relaxed requirement from the kernel regression provides flexibility and robustness to model misspecification. Another key advantage of introducing the kernel function is its ability to capture nonlinear relationships between the phenotype (***Y***) and the metabolome (***Z***) in a computationally tractable manner.

These relationships are assumed to exist in some feature space that is generated by a positive definite kernel function *K*(·,·). The kernel function can be understood as a feature map that delivers the dot product between *z*_*i*_ and *z*_*j*_ within the features space, i.e., *K*(*z*_*i*_, *z*_*j*_) = ⟨*ϕ*(*z*_*i*_),*ϕ*(*z*_*j*_)⟩, where *ϕ*(·) is the transformation to the feature space and ⟨·,·⟩ is the dot product. The representer theorem allows *h*(***Z***) to be represented through the kernel function *K*(·,·) as $h(\cdot )=\sum_{i=1}^{n}\alpha_{i}K(\cdot ,z_{i},\rho )$ for some coefficients *α*_*i*_∈ℝ. More detailed derivations can be found in texts by Schölkopf and Smola[52] as well as Cristianini and Shawe-Taylor[53].

The kernel function *K* can be thought of as a measurement of similarity between two individuals. Common choices for kernel functions are the *Linear Kernel:* $K(z_{i},z_{j})=z_{i}^{T}z_{j}$ (the dot product), the *dth Polynomial Kernel*: $K(z_{i},z_{j},\rho )={\left(z_{i}^{T}z_{j}+\rho \right)}^{d}$, and the *Gaussian Kernel*: *K*(*z*_*i*_, *z*_*j*_, *ρ*) = exp{−‖*z*_*i*_−*z*_*j*_‖^2^/*ρ*}, where ‖·‖ is the Euclidean (*L*_2_) norm. For this work, we employ the Gaussian kernel and use the median of all pairwise Euclidean distances between all *z*_*i*_ and *z*_*j*_ as an empirical estimate of *ρ*. We choose to work with the Gaussian kernel since it is a *characteristic* kernel, a desirable property meaning that probability measures embedded through the kernel function are unique.

#### Kernel-based score test

Liu et al. show a connection between kernel machine regression and linear mixed models for semiparametric modeling of high dimensional data [11,12]. The parameters ***β*** and *h*(***Z***) can be estimated by maximizing the scale penalized likelihood

$$L(\beta ,h)=-\frac{1}{2}\sum_{i=1}^{n}{\left[y_{i}-x_{i}^{T}\beta -h(z_{i})\right]}^{2}-\frac{1}{2}\lambda {\left\|h\right\|}^{2}$$ 2)

$$=-\frac{1}{2}\sum_{i=1}^{n}{\left[y_{i}-x_{i}^{T}\beta -\sum_{j=1}^{n}\alpha_{j}K(z_{i},z_{j})\right]}^{2}-\frac{1}{2}\lambda \alpha^{T}K\alpha ,$$ 3)

where ***K*** = *K*(*z*_*i*_, *z*_*j*_, *ρ*) is the semi-positive definite kernel function of choice. *h*(***Z***) can then be understood as subject specific random effects with mean 0 and variance *τ****K***. Testing for an association between phenotype and pathway is then equivalent to testing the null hypothesis *H*_0_:*τ* = 0 vs *H*_1_:*τ*>0. We adopt Chen et al.’s adjusted kernel association test adjusted for small samples, which is common for many omics studies [23]. The standard quadratic score statistic for kernel association tests,

$$Q(\beta ,\sigma ,\rho )=\frac{1}{\sigma^{2}}{\left(Y-X\beta \right)}^{T}K(Y-X\beta ),$$ 4)

is adjusted to account for the high variability in estimates of *σ*^2^ when *n* is small. The distribution of *Q* under the null model is then approximated as a weighted sum of *χ*^2^ variables using Davies method [27].

### Graph laplacian

A network or graph, *G* = {*V*, *E*}, is a mathematical representation of any interconnected structure through a set *V* of *p* nodes (or vertices) and a set *E* of edges, where the elements of *E* are pairs {*u*, *v*} of distinct vertices, *u*, *v*∈*V*. When applied to omic pathways, nodes represent individual metabolites, genes, microbes, etc. within the pathway and edges represent direct interactions/reactions between them.

Two important features of any graph are its adjacency matrix, ***A***, and degree matrix, ***D***. ***A*** is a *p*×*p* matrix that is non-zero when an edge exists between two vertices. ***D*** is a *p*×*p* diagonal matrix with ***D***_[*i*,*i*]_ representing the number of nodes connected to node *i*. For this work, we represent pathways using undirected unweighted graphs, i.e., there is no ordering to the vertices defining an edge and {*u*, *v*} = {*v*, *u*}∈*E*. This means ***A*** will be a symmetric matrix with all entries either 1 or 0. Using these features, we can calculate a graph’s *Laplacian* ***L***≔***D***−***A*** and its *normalized Laplacian* L˜≔D−12LD−12=I−D−12AD−12, where ***I*** is a *p*×*p* identity matrix.

Both ***L*** and $\tilde{L}$ can be regarded as linear operators of functions ***f***:*V*→ℝ that induce a semi-norm ‖***f***‖_*L*_ = ⟨***f*,*Lf***⟩ = ***f***^*T*^***Lf***. This semi-norm can be interpreted as a measure of “smoothness” or how much ***f*** varies over its domain. Standardizing ***L*** by the number of connections per node to obtain $\tilde{L}$ is a common approach in graph theory since $\tilde{L}$ has several well-known and desirable properties. In particular, $\tilde{L}$ is symmetric and positive semidefinite, and its eigenvalues, *λ*_*i*_, are bounded such that they satisfy 0≤*λ*_*i*_≤2 for *i*∈1,2,…*p*. Another interesting feature of a graph’s normalized Laplacian, $\tilde{L}$, is that the number of disjoint pieces within a graph is captured by the number of $\tilde{L}’s$ eigen values equal to 0 [54].

### Graph regularization

A key component of PaIRKAT is the ability to handle missing and incorrect information from the graph. Pathway databases may not be complete, and untargeted data generating techniques may not be able capture all components within a pathway. This leaves some pathways with low connectivity and others with completely disconnected nodes. This can lead to a decrease in our power to detect associations between phenotypes and metabolomic pathways. One proposed solution is to simply manipulate the adjacency matrix by adding a small constant to all entries[17,18], i.e. working with a modified adjacency matrix $\tilde{A}=A+tee^{\prime }$, where *t* is a nonnegative tuning parameter and *e* is a vector of 1s. This yields a full rank matrix as desired, but we know that the subspace spanned by $\tilde{A}$ is not the correct subspace on which our graph lies.

A more elegant solution can be drawn from Smola and Kondor’s work on regularization of graphs[25] in which they draw on parallels between the standard Laplacian operator $\left(\Delta =\frac{\partial^{2}}{\partial x_{1}^{2}}+\frac{\partial^{2}}{\partial x_{2}^{2}}+\cdots +\frac{\partial^{2}}{\partial x_{m}^{2}}\right)$ and the graph Laplacian to design regularization kernels for graphs. Rapaport, et al.[26] took a similar approach to graph smoothing, though this work was done in the context of classification not hypothesis testing. These ideas can be generalized further to represent any metric on a space. That is, for any two observations *i* and *j*, the inner product can be expressed as ${\left\langle z_{i},z_{j}\right\rangle }_{M}=z_{i}^{T}Mz_{j}$, where ***M*** defines the metric on the vector space based on ‖***z***_*i*_−***z***_*j*_‖_***M***_. Purdom[55] presents this argument in the context of a “generalized” principal component analysis using a general metric ***M***. This can be seen as an application of a linear kernel on any metric space, whereas we apply the Gaussian kernels for hypothesis testing and, like Rapaport, focus on graph Laplacians for our metric.

For this work, we apply a regularization function to obtain a *regularized* normalized Laplacian: $r(\tilde{L})\equiv {\tilde{L}}_{R}$. Regularizations of the Laplacian can be seen as regularizations of the eigenvalues of $\tilde{L}$, *r*(*λ*). There are many possible choices for *r*; the only requirement is that *r*^−1^(*λ*)>0 for *λ*∈[0, 2] to ensure $r(\tilde{L})≽0$. In classical Fourier analysis the size of *λ*_*i*_∈[0, 2] is directly proportional to the frequency of component *i* within Fourier space, which translates to the degree of noise within the system. This intuition tells us to limit *r*^−1^(*λ*) to monotonically increasing functions in order to impose higher penalties to more uneven portions of the graph while preserving the lower frequency components, which we assume translate to the prevalent biological signals. Smola and Kondor recommend further limiting choices of *r* to functions expressible by power series such as a *diffusion* kernel, $r(\tilde{L})=e^{-\tau /2\tilde{L}}$. See [56] for complete details on the derivation of different regularization functions.

PaIRKAT implements a “linear” regularization function

$${\tilde{L}}_{R}={\left(I+\tau \tilde{L}\right)}^{-1},$$ 5)

where *τ*>0 is a bandwidth parameter and *I* is a *p*×*p* identity matrix. We choose this regularization for its simplicity and interpretability of *τ*. Increasing *τ* linearly increases the amount of smoothing performed in *r*^−1^(*λ*) = 1+*τλ*. We can now conduct a kernel machine test while incorporating connectivity within a pathway through ${\tilde{L}}_{R}$ into (1) as

$$g(Y)=X\beta +h(Z{\tilde{L}}_{R})+\epsilon ,$$ 6)

where *h* is a kernel function applied to $Z{\tilde{L}}_{R}$ and the other model components are as described in (1). $Z{\tilde{L}}_{R}$ is changing ***Z***’s basis function to one defined by the Laplacian, with the new basis vectors representing noise dampened through the regularization function. This can be interpreted as transforming each subject’s phenotype to a weighted sum of each element where the weights are the elements’ proximity to each other within the pathway. This falls under the ‘guilt by association’ framework as nodes closer to each other will share more information and disconnected nodes will share none. The kernel-based score test can then be applied to obtain powerful tests for associations between connected or disconnected pathways and a phenotype of interest.

### Simulation study

#### Simulation scenarios

**Fig 4:**
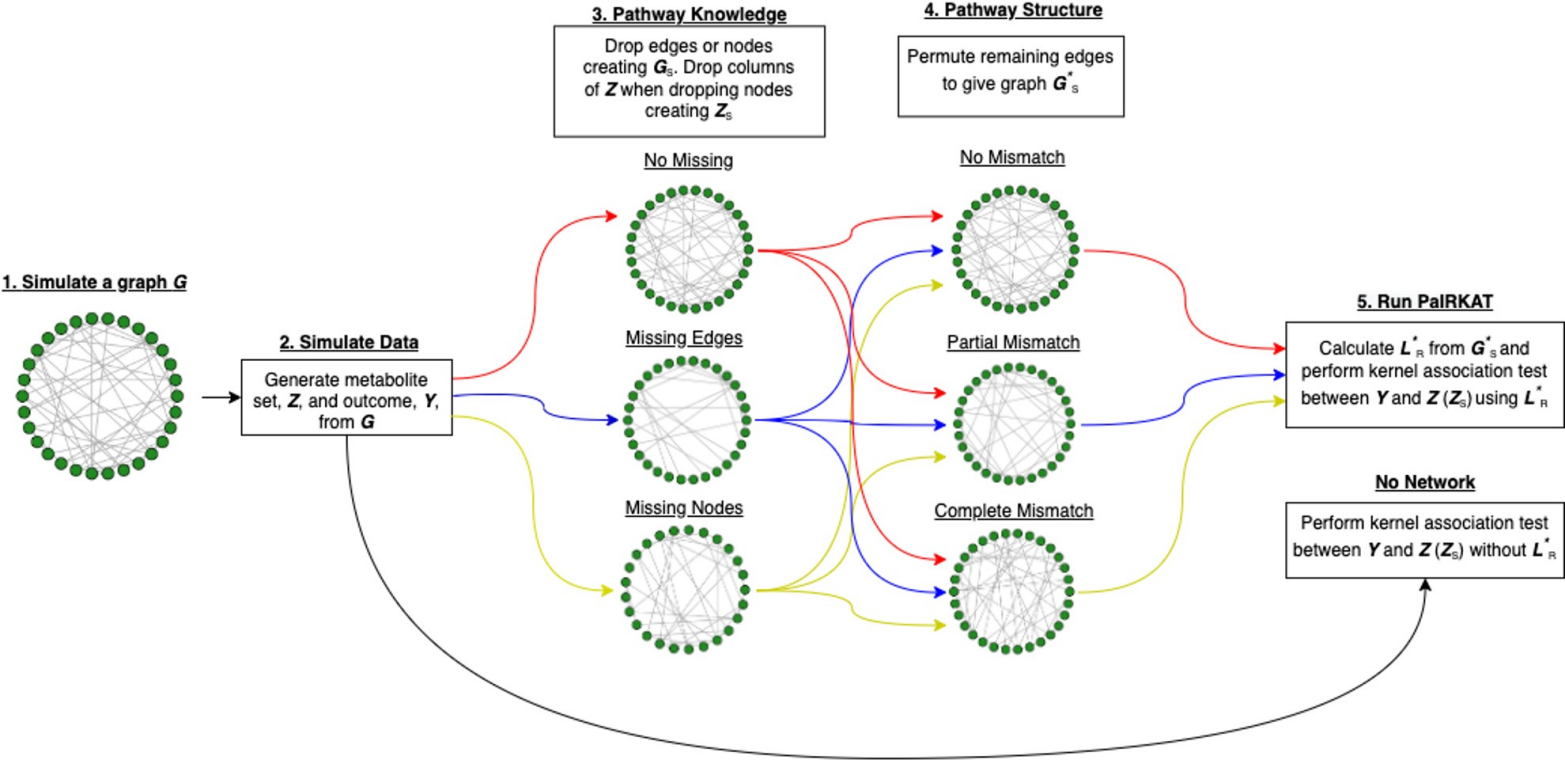
Flowchart of simulation procedure. We (1) simulate a graph *G*, (2) generate ***Z*** and ***Y*** from *G*, (3) drop nodes or edges from *G* to give a smaller graph *G*_*s*_ (drop corresponding columns of ***Z*** when dropping edges to create ***Z***_*s*_), (4) permute edges to create an improperly structured graph $G_{s}^{*}$, (5) calculate the regularized normalized Laplacian ${\tilde{L}}_{R}^{*}$ from $G_{s}^{*}$, and finally (5) test for an association between $h(Z\;{\tilde{L}}_{R}^{*})$ (or $h(Z_{s}\;{\tilde{L}}_{R}^{*})$) and ***Y*** in the model $Y=\beta_{0}+h(Z_{s}\;{\tilde{L}}_{R}^{*})$. For the “no network” simulations, we only use step (1), step (2) and step (5) without including ${\tilde{L}}_{R}^{*}$.

We conducted multiple simulation studies to assess whether the proposed method is robust to imperfect pathway information. We assumed 3 different “pathway knowledge” scenarios and 4 different “pathway structure” scenarios (Fig 4). Different pathway knowledge scenarios refer to different types of missing information, whereas pathway structure scenarios refer to different configurations of the “known” nodes and edges. We simulate using both the normalized Laplacian, $\tilde{L}$, and PaIRKAT’s regularized normalized Laplacian, ${\tilde{L}}_{R}$, as well as ignoring the pathway information. For comparison, we also tested using an F-test on all principal components of ***Z*** and $Z{\tilde{L}}_{R}$ and the minimum Simes’ adjusted p-value of univariate tests [29] on all columns of ***Z*** and $Z{\tilde{L}}_{R}$.

#### Pathway knowledge

We simulated three different *knowledge* scenarios to represent incomplete pathway database information and/or incomplete data collection.

1. No missing: Assuming the nodes measured (metabolites, genes, etc.) and edges connecting them are a perfect representation of the biological pathway of interest.
2. Missing edges: Assuming that some biological interactions (edges) are missing from the documented pathway. Here we generate a graph *G* = {*V*, *E*} according to the Barabasi-Albert model for a “low” edge density. We then give every set {*u*, *v*}∉*E* a 5% or 15% percent chance of being added to *E* for a “medium” or “high” edge density graph, respectively. ***Z*** and ***Y*** are then generated from the medium or high edge density graph, but $\tilde{L}$ or ${\tilde{L}}_{R}$ is calculated from the original “low” edge density graph. Examples of these graphs are shown in S3 Fig.
3. Missing nodes: Assuming that some of the nodes (and hence their edges) are missing from the documented pathway. Here a graph is used to generate ***Z*** and ***Y***. Then nodes with degree below the 25^th^ percentile have a 25% chance of being removed before calculating $\tilde{L}$ or ${\tilde{L}}_{R}$. The corresponding columns and rows of ***Z*** and $\vec{\beta }$ are removed as well. Examples of these graphs are shown in S4 Fig.

#### Pathway structures

**Fig 5:**
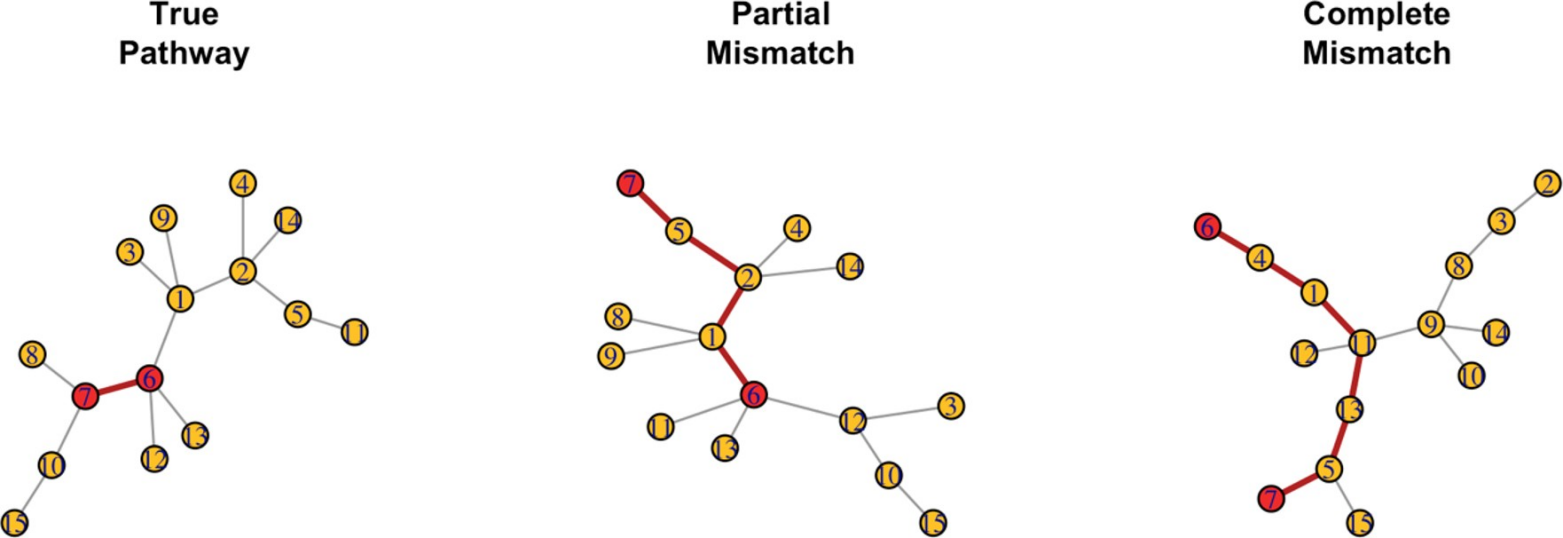
Examples of the three different pathway *structures*. Nodes 6 and 7 are highlighted in red to help display the effects of different pathway structures. (Left) The “true” pathway or graph that is used to simulated ***Z*** and ***Y***. This is the graph used for tests under a “perfect pathway structure” scenario. (Middle) A graph with approximately 40% of the edges from the “true” graph directly connecting the wrong nodes. This is used for tests under a “partial mismatch (40) structure” scenario. (Right) A graph with 0 shared edges with the “true” graph. This is the graph used for tests under a “complete mismatch structure” scenario.

After we simulate a pathway knowledge scenario, we alter the pathway *structure* to represent incorrect edge connections within a database. Examples of structures 1, 2, and 3 are displayed in Fig 5.

1. No mismatch: No alterations to graph edges. The graph used to simulate ***Z*** and ***Y*** is the same graph used to calculate $\tilde{L}$ or ${\tilde{L}}_{R}$ (Fig 5, left).
2. Partial Mismatch: a graph, *G*_1_ = {*V*_1_, *E*_1_}, is used to simulate ***Z*** and ***Y***. This graph’s edges are permuted such that any edge {*u*, *v*}∈*E*_1_ has a 10%, 40%, or 70% chance of being changed to some {*u*, *w*}∉*E*_1_; i.e., approximately 10%, 40%, or 70% of direct edges are incorrect before calculating $\tilde{L}$ or ${\tilde{L}}_{R}$ (Fig 5, middle).
3. Complete Mismatch: a network *G*_1_ = {*V*_1_, *E*_1_} is used to simulate ***Z*** and ***Y***. A new random graph, *G*_2_, is then draw and forced to have no edges that match *G*_1_, i.e., *V*_1_ = *V*_2_ but if {*u*, *v*}∈*E*_1_ then {*u*, *v*}∉*E*_2_. We then calculate $\tilde{L}$ or ${\tilde{L}}_{R}$ from *G*_2_ (Fig 5, right).
4. No Pathway: a graph is used to simulate ***Z*** and ***Y***. This connectivity is ignored while testing by not including $\tilde{L}$ or ${\tilde{L}}_{R}$ in the kernel function.

All pathway *structures* were considered under each different pathway *knowledge* scenario. The different pathway structures were imposed after simulating under different pathway knowledge assumptions. Each simulated pathway structure and knowledge combination followed 5 steps: (1) simulate a graph *G*, (2) generate ***Z*** and ***Y*** from *G*, (3) drop nodes and/or edges (based on *knowledge* assumption) from *G* and ***Z*** to give a smaller graph and node set *G*_*S*_ and ***Z***_*S*_, (4) alter *G*_*S*_ (based on *structure* assumption) to create a graph $G_{s}^{*}$ with improper edge connections, (5) calculate ${\tilde{L}}^{*}$ or ${\tilde{L}}_{R}^{*}$ from $G_{s}^{*}$, and (5) test for an association between $h(Z_{s}\;{\tilde{L}}_{R}^{*})$ and ***Y*** in the model $Y=\beta_{0}+h(Z_{s}\;{\tilde{L}}_{R}^{*})$. See Fig 4 for a flowchart of these simulation scenarios.

#### Simulated data

To evaluate PaIRKAT’s overall testing performance and robustness to incorrect pathway information, we simulate data and tests assuming various types of misspecified pathways. All simulations were performed using R[57]. Random graphs were generated using the *igraph*[58] package according to the Barabasi-Albert model[59] with *p* nodes representing *p* metabolites within a pathway. The graph’s adjacency matrix was converted into a positive definite precision matrix, **Ω**, using an approached developed by Danaher, et al.[60] and also applied by Shaddox, et al[61]. An *n* by *p* matrix of metabolite abundances, ***Z***, was then simulated from a multivariate normal distribution with mean **0** and covariance **Ω**^−1^. In this way, node connectivity is captured by **Ω**. A continuous outcome *Y*_*i*_ was then simulated from a normal distribution with mean 0.26+0.5 X_1_+0.25 X_2_+∑_*j*_*β*_*j*_*Z*_*ij*_ and variance *σ*^2^, where X_1_ was a binary variable, X_2_ is a uniform random variable, *σ*^2^ = 1.3688^2^. This value for *σ*^2^ was drawn from observed metabolomics data. The regularization parameter *τ* is set to 1 for all simulations. All *β*_*j*_ were set to 0 to assess Type I error rates or set to 0.1 to assess power for the different pathway information scenarios described above. Each used 10,000 simulations of graphs of size *p* = 15, 30, 45 assuming a sample size of *n* = 160, and a testing level of *α* = 0.05 was used for all simulations.

### COPDGene data

We analyzed data collected from the COPDGene study [22], a multicenter observational study that collected genetic data as well as multiple measures of lung function to study chronic obstructive pulmonary disease (COPD). Between 2007 and 2011, 10,198 participants with and without chronic obstructive pulmonary disease (COPD) enrolled (Visit 1). A five-year follow up visit took place between 2013 and 2017 (Visit 2). Blood samples were also obtained for -omics analyses from participants who provided consent. In total, 1136 subjects (1040 non-Hispanic white, 96 African American) participated in a metabolomics ancillary study in which they provide fresh frozen plasma collected using an 8.5 mL p100 tube (Becton Dickson) at Visit 2.

### Metabolomics and data processing

P100 plasma was profiled using the Metabolon (Durham, NC, USA) Global Metabolomics platform. Briefly, untargeted liquid chromatography–tandem mass spectrometry (LC–MS/MS) was used to quantify 1392 metabolites and described in[62,63]. A data normalization step was performed to correct variation resulting from instrument inter-day tuning differences: metabolite intensities were divided by the metabolite run day median, then multiplied by the overall metabolite median. It was determined that no further normalization was necessary based on the reduction in the significance of association between the top PCs and sample run day after normalization. Subjects with aggregate metabolite median *z*-scores greater than 3.5 standard deviation from the mean (*n* = 6) of the cohort were removed. Metabolites were excluded if >20% of samples were missing values[64]. For the 995 remaining metabolites, missing values were imputed across metabolites with k-nearest neighbors imputation (*k* = 10) using the R package *impute*[65]. As a final step, metabolomic data was natural log transformed and standardized. Linear regression models were fit to each metabolite controlling for white blood cell count, percent eosinophil, percent lymphocytes, percent monocytes, percent neutrophils, and hemoglobin. The partial residuals were then used as the observed metabolomics data. These data are available at Metabolomics Workbench with identifier PR000907.

Four hundred and thirty six of these metabolites had an id in the KEGG database of human pathways, which was accessed using the *keggLink* function from the *KEGGREST* package[66]. These 436 metabolites appear in 161 KEGG pathways, and 28 of these 161 KEGG pathways contained 10 or more metabolites. Edges in a pathway’s graph were defined by connections within a pathway from the KEGG reaction database. Note that our filtered dataset did not contain every metabolite within the 28 KEGG pathways selected, and therefore some of the analyzed pathways have less that 10 metabolites.

### Clinical variables

We focus on two COPD phenotypes: (1) percent emphysema and (2) the ratio of post-bronchodilator forced expiratory volume at one second divided by forced vital capacity (FEV_1_/FVC). Emphysema, a measure of erosion of the distal airspaces, has been linked with the clinical severity of COPD[67]. It is an imaging-based phenotype defined as the 15th percentile lung voxel density in Hounsfield units adjusted for total lung capacity from quantitative CT imaging analyses. FEV_1_/FVC is a measure of airflow obstruction. To normalize FEV_1_/FVC, we use the following log ratio transformation, $log\;\left(\frac{FEV_{1}/FVC}{1-FEV_{1}/FVC}\right)$. After removing incomplete cases we were left with 1,113 complete cases for the FEV_1_/FVC analysis and 1,065 complete cases for the percent emphysema analysis.

### Analysis

We compared results from tests that included pathway connectivity via $\tilde{L},\;{\tilde{L}}_{R},$ and tests that ignored pathway connectivity for the 28 pathways that had measurements on at least 10 of the metabolites in the pathway. P-values were calculated from a score test as described Section 2 with *τ* = 1 for PaIRKAT tests. P-values from each method were indistinguishable from one another for both data sets with over 1,000 observations. However, many data sets may not be that large. To demonstrate the differences in performance, 100 random subsets of sizes 100, 200, 300, 400, and 500 were taken from both the log FEV_1_/FVC ratio and the percent emphysema data sets. All three methods were used to test for associations between phenotype and metabolites within a pathway. The 100 p-values were then averaged to measure the performance of each method. All null models included subject age, sex, BMI, smoking status (current, former, never), pack-years of smoking, and the clinical center as covariates.

## Supporting information

S1 FigAssociations between metabolite subsets and log FEV1/FVC ratio.Average p-values from kernel regressing tests that do not include pathway information (No Laplacian, red circles), include pathway information through a normalized Laplacian ($\tilde{L}$, green triangles), and include pathway information through a regularized normalized Laplacian (${\tilde{L}}_{R}={\left(I+\tau \tilde{L}\right)}^{-1}$, blue squares) are displayed. P-values were averaged over 100 random subsets of size 100, 200, 300, 400, and 500 from the COPDGene dataset. *τ* was set to 1 for all tests that used ${\tilde{L}}_{R}$.(TIF)

S2 FigAssociations between metabolite subsets and percent emphysema.Average p-values from kernel regressing tests that do not include pathway information (No Laplacian, red circles), include pathway information through a normalized Laplacian ($\tilde{L}$, green triangles), and include pathway information through a regularized normalized Laplacian (${\tilde{L}}_{R}={\left(I+\tau \tilde{L}\right)}^{-1}$, blue squares) are displayed. P-values were averaged over 100 random subsets of size 100, 200, 300, 400, and 500 from the COPDGene dataset. *τ* was set to 1 for all tests that used ${\tilde{L}}_{R}$.(TIF)

S3 Fig Examples graphs with high, medium, and low edge densities. Low density graphs were generated according the Barabasi-Albert model for graph simulation. Medium- and high-density graphs were generated by giving each unconnected node either a 5% or 15% chance of becoming connected, respectively.
(TIF)

S4 Fig Example of a graph with missing nodes. Graphs were generated according to the Barabasi-Albert model. Then any node with degree below the 25^th^ percentile of degrees within the graph had a 25% chance of being dropped.
(TIF)

S1 TableType 1 error rates using complete pathway.Error rates were calculated from score tests on 1000 simulated data sets. All simulations used graphs with 15, 30, or 45 nodes. No nodes or edges were dropped for these simulations. Pathway information was included in kernel score test through the normalized Laplacian $\tilde{L}$.(XLSX)

S2 TableType 1 error rates using pathways with 5% missing edges.Error rates were calculated from score tests on 1000 simulated data sets using graphs with 15, 30, or 45 nodes. The graph used to simulate ***Z*** and ***Y*** was of medium edge density, while the graph used to test was of low density. The low-density graphs are drawn from the Barabasi-Albert model with edge density 0.13, 0.07, and 0.04 for graphs with 15, 30, and 45 nodes, respectively. Medium edge density graphs are created by giving any 2 nodes without a direct edge between them a 5% chance of becoming directly connected. This creates graphs with an average edge density of 0.18, 0.11, and 0.09 for graphs with 15, 30, and 45 nodes, respectively. Pathway information was included in kernel score test through the normalized Laplacian $\tilde{L}$.(XLSX)

S3 TableType 1 error rates using pathways with 15% missing edges.Error rates were calculated from score tests on 1000 simulated data sets using graphs with 15, 30, or 45 nodes. The graph used to simulate ***Z*** and ***Y*** was of high edge density, while the graph used to test was of low density. The low density graphs are drawn from the Barabasi-Albert model with edge density 0.13, 0.07, and 0.04 for graphs with 15, 30, and 45 nodes, respectively. High edge density graphs are created by giving any 2 nodes without a direct edge between them a 15% chance of becoming directly connected. This creates graphs with an average edge density of 0.26, 0.21, and 0.19 for graphs with 15, 30, and 45 nodes, respectively. Pathway information was included in kernel score test through the normalized Laplacian $\tilde{L}$.(XLSX)

S4 TableType 1 error rates using pathways with dropped nodes.Error rates were calculated from score tests on 1000 simulated data sets using graphs 15, 30, or 45 nodes initially. The graph used to simulate ***Z*** and ***Y*** contained all nodes. Nodes with degree below the 25^th^ percentile within a graph had a 25% chance of being dropped before testing. Pathway information was included in kernel score test through the normalized Laplacian $\tilde{L}$.(XLSX)
