# Supplementary figures and images for: PaIRKAT: A pathway integrated regression-based kernel association test with applications to metabolomics and COPD phenotypes
Source: PLoS Comput Biol. 2021 Oct 22;17(10):e1008986. doi: 10.1371/journal.pcbi.1008986 (PMC8565741; doi:10.1371/journal.pcbi.1008986)

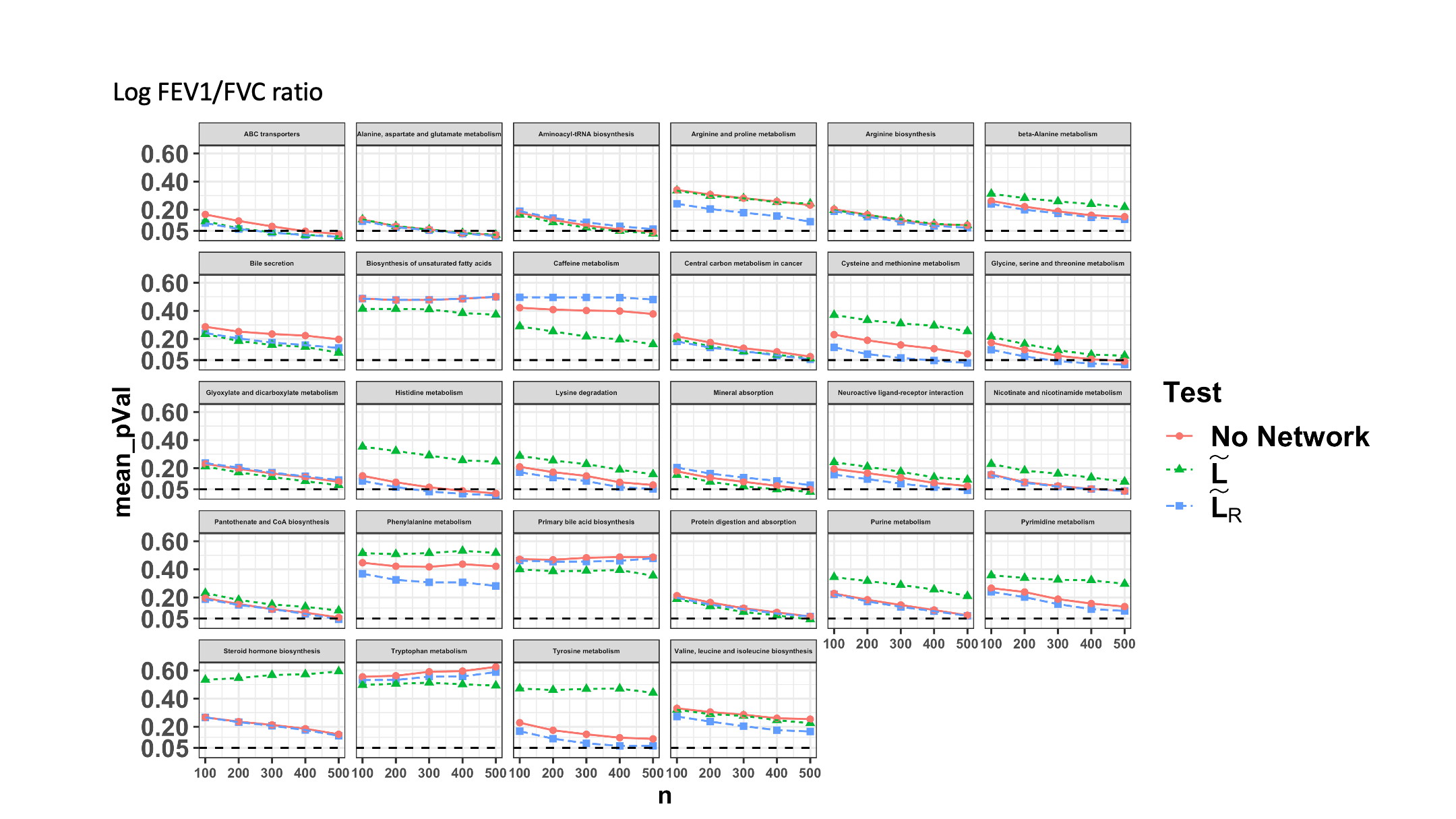

Supplement: S1 Fig — Average p-values from kernel regressing tests that do not include pathway information (No Laplacian, red circles), include pathway information through a normalized Laplacian (L˜, green triangles), and include pathway information through a regularized normalized Laplacian (L˜R=(I+τL˜)−1, blue squares) are displayed. P-values were averaged over 100 random subsets of size 100, 200, 300, 400, and 500 from the COPDGene dataset. τ was set to 1 for all tests that used L˜R. (TIF) [file pcbi.1008986.s001.tif]

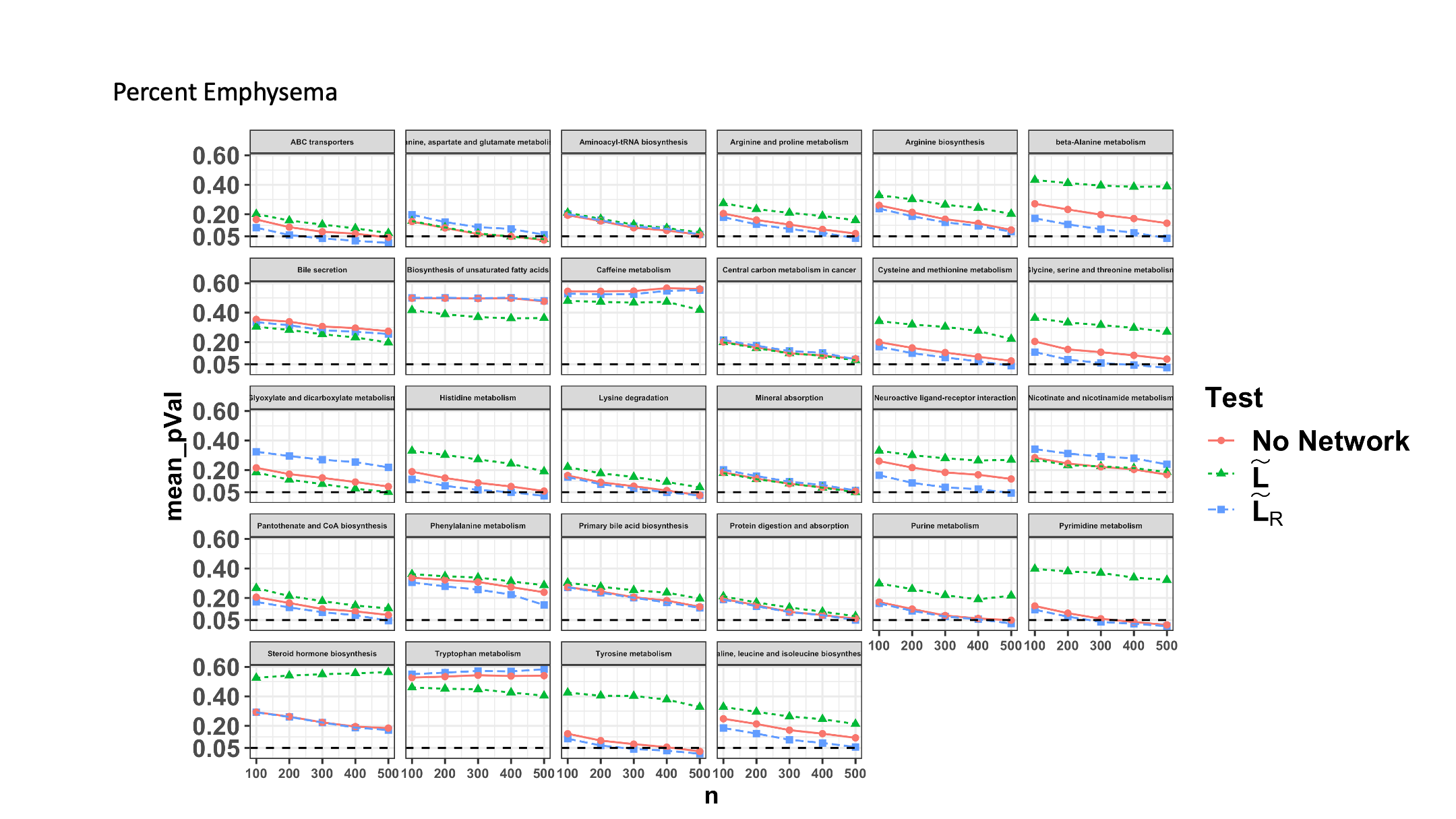

Supplement: S2 Fig — Average p-values from kernel regressing tests that do not include pathway information (No Laplacian, red circles), include pathway information through a normalized Laplacian (L˜, green triangles), and include pathway information through a regularized normalized Laplacian (L˜R=(I+τL˜)−1, blue squares) are displayed. P-values were averaged over 100 random subsets of size 100, 200, 300, 400, and 500 from the COPDGene dataset. τ was set to 1 for all tests that used L˜R. (TIF) [file pcbi.1008986.s002.tif]

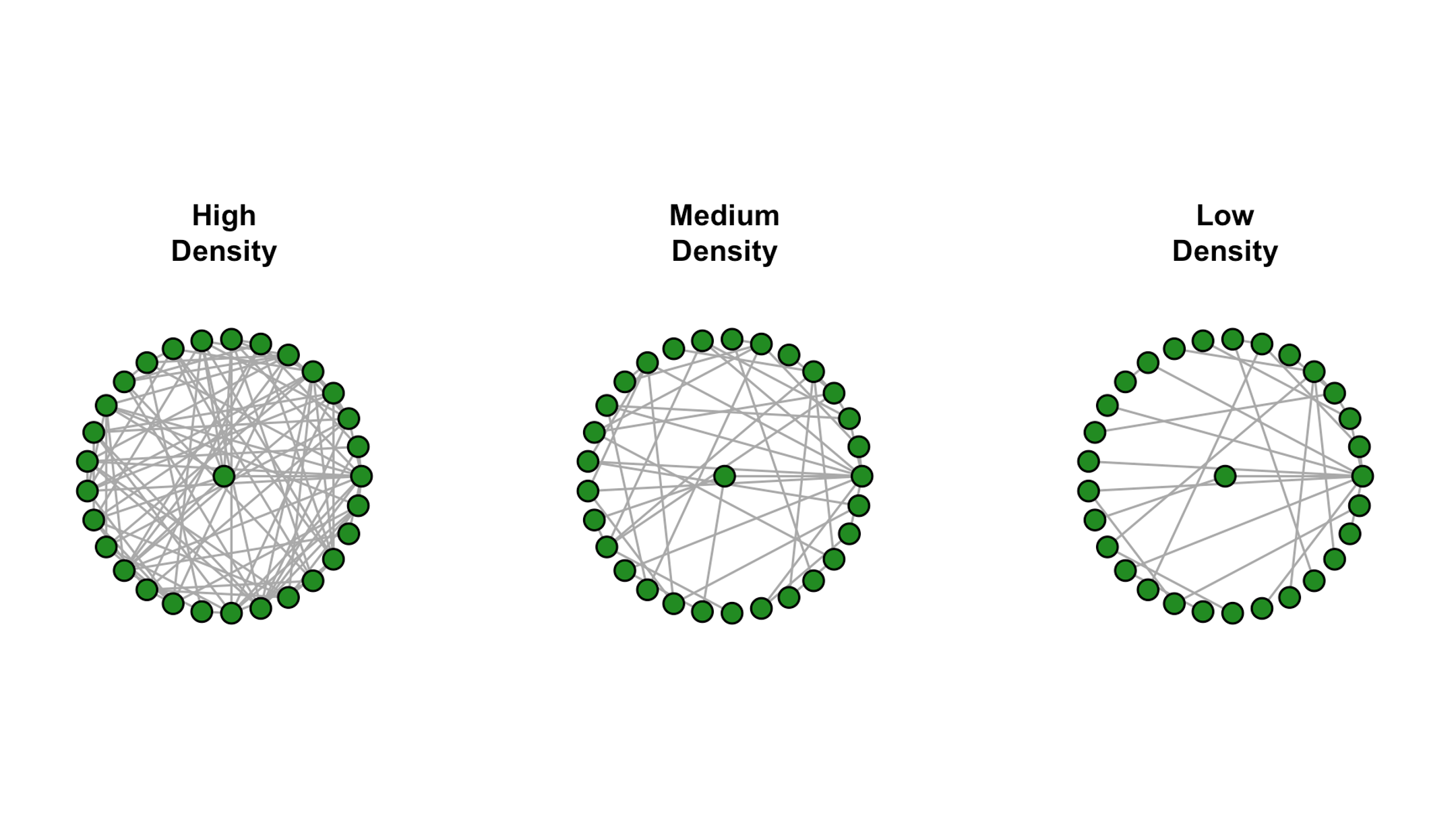

Supplement: S3 Fig — Low density graphs were generated according the Barabasi-Albert model for graph simulation. Medium- and high-density graphs were generated by giving each unconnected node either a 5% or 15% chance of becoming connected, respectively. (TIF) [file pcbi.1008986.s003.tif]

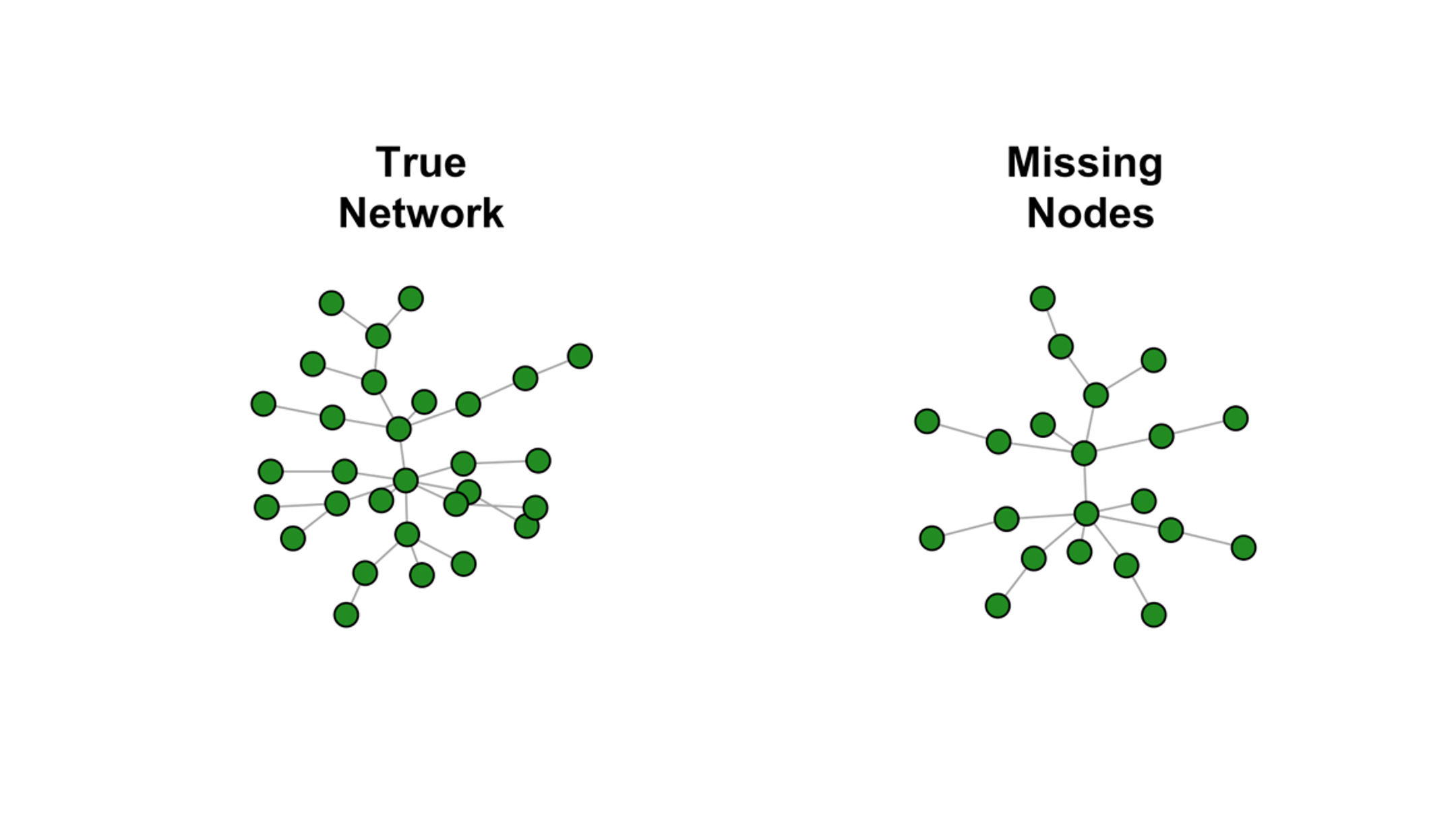

Supplement: S4 Fig — Graphs were generated according to the Barabasi-Albert model. Then any node with degree below the 25th percentile of degrees within the graph had a 25% chance of being dropped. (TIF) [file pcbi.1008986.s004.tif]
