# Supplementary material for: PaIRKAT: A pathway integrated regression-based kernel association test with applications to metabolomics and COPD phenotypes
Source: PLoS Comput Biol. 2021 Oct 22;17(10):e1008986. doi: 10.1371/journal.pcbi.1008986 (PMC8565741; doi:10.1371/journal.pcbi.1008986)
Supplement: Attachment — Submitted filename: PaIRKAT_Rebuttal.docx [file pcbi.1008986.s009.docx]

Reviewer's Responses to Questions

**Comments to the Authors:
Please note here if the review is uploaded as an attachment.**

Reviewer #1: In this paper the authors proposed a novel approach for pathway-based kernel test with application to metabolomics data. A new idea is to use a smoothed graph Laplacian matrix based on a likely incomplete graph or network for some metabolites (or genes or …) in a pathway. Through simulated and real data examples, the new test was shown to improve over using a standard graph Laplacian and not using any graph/network information. The proposed method has potential to be useful in practice.

Main comments.
1. Would the author explain why the proposed method works? In particular, the main idea is to replace h(Z) by h(ZL_R) in eq (6); why?

We have added a short explanation in line 140 and a fuller discussion to lines 478-484.

2. I am really surprised by how poorly using the standard Laplacian L performed in Figs 1 and 2, much worse than not using any network information. On the other hand, Presumably, all the information from L_R is more or less from L. How to explain this? Did you just replace h(Z) by h(ZL) in eq (6)? If so, then there may be a problem: By eq (5), it seems that L_R is in the scale of the (generalized) inverse of L, so perhaps a generalized inverse of L should be used?

You are correct, we replace h(Z) with h(ZL) in eq (6) for our normalized Laplacian simulations and tests. You are also correct that a generalized inverse would solve the problem of L being singular (determinant will always be 0 since at least one eigen value is 0). However, this does not accomplish our goal of dampening out the noise contained within L while preserving important structural information from the pathway. The regularization function we apply to the eigen values (i.e., r(lambda)) is an important piece of the method with a theoretical justification and has been shown to work in practice (Smola and Kondor 2003, Rapaport 2006). You are also correct to point out that it is a very small adjustment to L, but we wanted to make sure not to ‘over smooth’ our graphs.

3. It would be useful to consider a situation when there is indeed NO information from a network/graph, how the proposed and other methods perform?

In this case, L would be an empty matrix (all entries = 0). Any normalization technique we apply would return the inverse of the identity matrix (the identity matrix), and we would not be changing our Z in eq (6). We can see how the approaches give very similar results in the left column of Figure 3 (only 1 edge in the graph).

4. In eq (5), how \tau was selected in your simulations and real data examples? What were its typical values?

Tau was set to 1 for all simulations and real data examples as we did not want to over-smooth the graph.

5. Table 1 Caption is hard to understand: if “No nodes or edges were dropped for these simulations”, what is the meanings of “Perfect” vs “Partial 10%”?

Thank you, this has been updated in all tables.

6. Author summary: “It is common for nodes (e.g. metabolites) to be disconnected from all others within the graph, which leads to meaningful decreases in testing power whether or not the graph information is included.”
i) If no graph info is used, why does it matter whether a node is connected or not with others?

This was poorly worded. Thank you for pointing it out. We meant to say, “meaningful decreases in testing power when graph information is ignored.” It has been updated.

ii) If in truth a node is NOT connected with other nodes, compared to what the power will decrease?

If a node is not connected to other nodes (i.e. the pathway is an incomplete graph), the kernel test has using h(Z) has much lower power than using h(Z*L_R).

Minor comments: There are a few statements I do not understand; below are some examples.
1. “Disjoint nodes are of concern for graph-structured data since many techniques for inference on graph-structured data involve spectral decomposition and require full rank matrices from a fully connected graph.” I am not sure many methods require this: e.g. most spectral graph-based methods do NOT require a Laplacian to be of full rank.
2. “This method and others like it are tailored to genomic data and not applicable to other omics data.” Why NOT, given that “Omics data (metabolomics, genomics, etc.) can often …”?

Thank you for the question. In the sentence “Omics data (metabolomics, genomics, etc.) can often …” we are referring to the common issues that come from p>>n, the need for imputation, the need for normalization, etc. I.e. more high level concerns.

The examples built for genes that we site draws information from SNP to gene mappings. For example, Freytag et al. 2013 use a matrix with 0s or 1s indicating whether or not SNPs belong to a gene sequence. Other omics (metabolomics, microbiome, etc.) has no analogous structure, so our method is more broadly applicable is this sense.

Reviewer #2: This paper concerns the development of a pathway based test for metabolomics data. The authors specifically choose to use a kernel testing framework and propose to incorporate known pathway structure into the test by prescaling the data by the graph Laplacian. The authors make particular emphasis on the situation in which there is sparsity in the data. Overall, the work seems reasonable and the authors have been quite thorough in their investigations. I particularly like the careful consideration of the impact of incorrect network structure. This has the potential to be a nice contribution to the literature. However, I do have a few comments which follow below in no particular order.

1. The type I error rate of the kernel test appears to be quite inflated in the tables, particularly for larger pathways. In many cases, the type I error rate is 0.06 which 20% above the nominal 0.05 level and probably worse at lower alpha levels. I believe that this inflated false positive rate may be partially due to the outdated Satterthwaite method used in the authors approach. The kernel testing literature has long left this approach behind. More recent developments include using the Davies approach, saddlepoint approximations, and other small sample approximations that work better with full rank kernels, e.g. the gaussian. These approaches should be incorporated to correct the unacceptably high type I error.

Thank you, the methods have been updated to use Davies method and an adjusted score statistic from Chen et al.

2. It would be helpful if the authors could provide additional insight as to *why* incorporation of prior knowledge (graph structure) is useful in this case. Work by Schaid (upon which Freytag builds, I recall) emphasizes the idea of “guilt by association”. Is that a similar idea here?

We have added a short explanation in line 140 and a fuller discussion to lines 478-484.

3. While kernel based approaches appear generally reasonable for this context. I think it important to note that the hypothesis in this case is a global null: that is, if even a single metabolite is associated with the outcome then the entire pathway would be considered significant (see Goeman and Buhlmann for a technical discussion). It should be noted that some investigators find that this violates the spirit of a pathway analysis, but I do not feel strongly here other than it should be noted.

Thank you for this point of discussion and corresponding reference. We have added it to the discussion.

4. The discussion of comparisons with 2-step approaches ignores the fact that the inherent hypotheses are different which is an important distinction. Notably, most 2-step enrichment approaches are statistically invalid (Goeman and Buhlmann).

Thank you for this correction. These discussions have been removed.

5. There is a distinct lack of comparison with competing methods. The paper is entirely kernel test centric without consideration for competing approaches. Simple strategies would be using the top PC(s), minimum p-value approaches, or tail strength strategies.

Thank you for your suggestions on appropriate competing methods. We have added power and Type I error simulations for F-tests using top PC(s) and taking the minimum Simes’ adjusted p-value as comparisons. We originally considered the “No Network” approaches as the competing method to beat.

6. Instead of using prior knowledge, I imagine that another strategy seems to be directly estimating network structure from the data, e.g. through the graphical Lasso. This approach is taken by Zhan et al. This strategy would be different in the sense that connectivity is not due to a particular biological cascade but rather correlation in the data. I would be curious to see how this performs

Thank you for your thoughts on this issue. It is an interesting question and was a discussion within our group as well. Our main concern was too many layers of data driven structures would lead to “overfitting” of a kind. Additionally, many data driven methods for network estimation (e.g. graphical Lasso, or pairwise correlation approaches) focus on linear relationships. This relationship is included within the Gaussian Kernel since it is one of the pieces of the Taylor’s expansion of *e*. Our thinking is the established biological interactions could truly be “additional” information.

We leave this exploration to further work but have added a few thoughts to the discussion.
