# Supplementary material for: PaIRKAT: A pathway integrated regression-based kernel association test with applications to metabolomics and COPD phenotypes
Source: PLoS Comput Biol. 2021 Oct 22;17(10):e1008986. doi: 10.1371/journal.pcbi.1008986 (PMC8565741; doi:10.1371/journal.pcbi.1008986)
Supplement: Attachment — Submitted filename: PaIRKAT_Re_Rebuttal.docx [file pcbi.1008986.s010.docx]

Reviewer's Responses to Questions

**Comments to the Authors:
Please note here if the review is uploaded as an attachment.**

Reviewer #2: This paper concerns the development of a pathway based test for metabolomics data. The authors specifically choose to use a kernel testing framework and propose to incorporate known pathway structure into the test by prescaling the data by the graph Laplacian. The authors make particular emphasis on the situation in which there is sparsity in the data. Overall, the work seems reasonable and the authors have been quite thorough in their investigations. I particularly like the careful consideration of the impact of incorrect network structure. This has the potential to be a nice contribution to the literature. However, I do have a few comments which follow below in no particular order.


2. It would be helpful if the authors could provide additional insight as to *why* incorporation of prior knowledge (graph structure) is useful in this case. Work by Schaid (upon which Freytag builds, I recall) emphasizes the idea of “guilt by association”. Is that a similar idea here?

We updated our manuscript to provide a more intuitive explanation of the method. We have expanded the background to include a larger discussion of Schaid and Freytag’s works in lines 110-115.

We have included how our method is similar to the work of Schaid and Freytag. We discuss how the idea of ‘guilty by association’ relate to our proposal in lines 149-156.

We also removed the more technical discussion from lines 482-487 and replaced it with a more direct discussion about how incorporating network information is related to information sharing based on the network architecture.
