# Supplementary material for: PaIRKAT: A pathway integrated regression-based kernel association test with applications to metabolomics and COPD phenotypes
Source: PLoS Comput Biol. 2021 Oct 22;17(10):e1008986. doi: 10.1371/journal.pcbi.1008986 (PMC8565741; doi:10.1371/journal.pcbi.1008986)
Supplement: Peer review history

# Peer review: Decision Letter 0

**DOI:** 10.1371/journal.pcbi.1008986.r001

**Authors:** Mark Alber, Carl Herrmann

30 May 2021

Dear Carpenter,

Thank you very much for submitting your manuscript "PaIRKAT: A pathway integrated regression-based kernel association test with applications to metabolomics and COPD phenotypes" for consideration at PLOS Computational Biology.

As with all papers reviewed by the journal, your manuscript was reviewed by members of the editorial board and by two independent reviewers. In light of the reviews (below this email), we would like to invite the resubmission of a significantly-revised version that takes into account the reviewers' comments.

As you will see, the reviewers find the method of potential interest, but havee raised several questions regarding the details of the methods, which you should clarify. One reviewer has also suggested some changes in the method, that we would ask you to consider, in order to handle the Type 1 error rate. In addition, comparison with alternative methods should be strengthen.

We cannot make any decision about publication until we have seen the revised manuscript and your response to the reviewers' comments. Your revised manuscript is also likely to be sent to reviewers for further evaluation.

When you are ready to resubmit, please upload the following:

[1] A letter containing a detailed list of your responses to the review comments and a description of the changes you have made in the manuscript. Please note while forming your response, if your article is accepted, you may have the opportunity to make the peer review history publicly available. The record will include editor decision letters (with reviews) and your responses to reviewer comments. If eligible, we will contact you to opt in or out.

[2] Two versions of the revised manuscript: one with either highlights or tracked changes denoting where the text has been changed; the other a clean version (uploaded as the manuscript file).

Important additional instructions are given below your reviewer comments.

Please prepare and submit your revised manuscript within 60 days. If you anticipate any delay, please let us know the expected resubmission date by replying to this email. Please note that revised manuscripts received after the 60-day due date may require evaluation and peer review similar to newly submitted manuscripts.

Thank you again for your submission. We hope that our editorial process has been constructive so far, and we welcome your feedback at any time. Please don't hesitate to contact us if you have any questions or comments.

Sincerely,

Carl Herrmann, Ph.D.

Associate Editor

PLOS Computational Biology

Mark Alber

Deputy Editor

PLOS Computational Biology

***********************

Reviewer's Responses to Questions

**Comments to the Authors:**

**Please note here if the review is uploaded as an attachment.**

Reviewer #1: In this paper the authors proposed a novel approach for pathway-based kernel test with application to metabolomics data. A new idea is to use a smoothed graph Laplacian matrix based on a likely incomplete graph or network for some metabolites (or genes or …) in a pathway. Through simulated and real data examples, the new test was shown to improve over using a standard graph Laplacian and not using any graph/network information. The proposed method has potential to be useful in practice.

Main comments.

1. Would the author explain why the proposed method works? In particular, the main idea is to replace h(Z) by h(ZL_R) in eq (6); why?

2. I am really surprised by how poorly using the standard Laplacian L performed in Figs 1 and 2, much worse than not using any network information. On the other hand, Presumably, all the information from L_R is more or less from L. How to explain this? Did you just replace h(Z) by h(ZL) in eq (6)? If so, then there may be a problem: By eq (5), it seems that L_R is in the scale of the (generalized) inverse of L, so perhaps a generalized inverse of L should be used?

3. It would be useful to consider a situation when there is indeed NO information from a network/graph, how the proposed and other methods perform?

4. In eq (5), how \\tau was selected in your simulations and real data examples? What were its typical values?

5. Table 1 Caption is hard to understand: if “No nodes or edges were dropped for these simulations”, what is the meanings of “Perfect” vs “Partial 10%”?

6. Author summary: “It is common for nodes (e.g. metabolites) to be disconnected from all others within the graph, which leads to meaningful decreases in testing power whether or not the graph information is included.”

i) If no graph info is used, why does it matter whether a node is connected or not with others?

ii) If in truth a node is NOT connected with other nodes, compared to what the power will decrease?

Minor comments: There are a few statements I do not understand; below are some examples.

1. “Disjoint nodes are of concern for graph-structured data since many techniques for inference on graph-structured data involve spectral decomposition and require full rank matrices from a fully connected graph.” I am not sure many methods require this: e.g. most spectral graph-based methods do NOT require a Laplacian to be of full rank.

2. “This method and others like it are tailored to genomic data and not applicable to other omics data.” Why NOT, given that “Omics data (metabolomics, genomics, etc.) can often …”?

Reviewer #2: This paper concerns the development of a pathway based test for metabolomics data. The authors specifically choose to use a kernel testing framework and propose to incorporate known pathway structure into the test by prescaling the data by the graph Laplacian. The authors make particular emphasis on the situation in which there is sparsity in the data. Overall, the work seems reasonable and the authors have been quite thorough in their investigations. I particularly like the careful consideration of the impact of incorrect network structure. This has the potential to be a nice contribution to the literature. However, I do have a few comments which follow below in no particular order.

1. The type I error rate of the kernel test appears to be quite inflated in the tables, particularly for larger pathways. In many cases, the type I error rate is 0.06 which 20% above the nominal 0.05 level and probably worse at lower alpha levels. I believe that this inflated false positive rate may be partially due to the outdated Satterthwaite method used in the authors approach. The kernel testing literature has long left this approach behind. More recent developments include using the Davies approach, saddlepoint approximations, and other small sample approximations that work better with full rank kernels, e.g. the gaussian. These approaches should be incorporated to correct the unacceptably high type I error.

2. It would be helpful if the authors could provide additional insight as to *why* incorporation of prior knowledge (graph structure) is useful in this case. Work by Schaid (upon which Freytag builds, I recall) emphasizes the idea of “guilt by association”. Is that a similar idea here?

3. While kernel based approaches appear generally reasonable for this context. I think it important to note that the hypothesis in this case is a global null: that is, if even a single metabolite is associated with the outcome then the entire pathway would be considered significant (see Goeman and Buhlmann for a technical discussion). It should be noted that some investigators find that this violates the spirit of a pathway analysis, but I do not feel strongly here other than it should be noted.

4. The discussion of comparisons with 2-step approaches ignores the fact that the inherent hypotheses are different which is an important distinction. Notably, most 2-step enrichment approaches are statistically invalid (Goeman and Buhlmann).

5. There is a distinct lack of comparison with competing methods. The paper is entirely kernel test centric without consideration for competing approaches. Simple strategies would be using the top PC(s), minimum p-value approaches, or tail strength strategies.

6. Instead of using prior knowledge, I imagine that another strategy seems to be directly estimating network structure from the data, e.g. through the graphical Lasso. This approach is taken by Zhan et al. This strategy would be different in the sense that connectivity is not due to a particular biological cascade but rather correlation in the data. I would be curious to see how this performs

**********

**Have the authors made all data and (if applicable) computational code underlying the findings in their manuscript fully available?**

The PLOS Data policy requires authors to make all data and code underlying the findings described in their manuscript fully available without restriction, with rare exception (please refer to the Data Availability Statement in the manuscript PDF file). The data and code should be provided as part of the manuscript or its supporting information, or deposited to a public repository. For example, in addition to summary statistics, the data points behind means, medians and variance measures should be available. If there are restrictions on publicly sharing data or code —e.g. participant privacy or use of data from a third party—those must be specified.

Reviewer #1: Yes

Reviewer #2: Yes

**********

PLOS authors have the option to publish the peer review history of their article (what does this mean?). If published, this will include your full peer review and any attached files.

If you choose “no”, your identity will remain anonymous but your review may still be made public.

**Do you want your identity to be public for this peer review?** For information about this choice, including consent withdrawal, please see our Privacy Policy.

Reviewer #1: No

Reviewer #2: No

Figure Files:

While revising your submission, please upload your figure files to the Preflight Analysis and Conversion Engine (PACE) digital diagnostic tool, https://pacev2.apexcovantage.com. PACE helps ensure that figures meet PLOS requirements. To use PACE, you must first register as a user. Then, login and navigate to the UPLOAD tab, where you will find detailed instructions on how to use the tool. If you encounter any issues or have any questions when using PACE, please email us at figures@plos.org.

Data Requirements:

Please note that, as a condition of publication, PLOS' data policy requires that you make available all data used to draw the conclusions outlined in your manuscript. Data must be deposited in an appropriate repository, included within the body of the manuscript, or uploaded as supporting information. This includes all numerical values that were used to generate graphs, histograms etc.. For an example in PLOS Biology see here: http://www.plosbiology.org/article/info%3Adoi%2F10.1371%2Fjournal.pbio.1001908#s5.

Reproducibility:

To enhance the reproducibility of your results, we recommend that you deposit your laboratory protocols in protocols.io, where a protocol can be assigned its own identifier (DOI) such that it can be cited independently in the future. Additionally, PLOS ONE offers an option to publish peer-reviewed clinical study protocols. Read more information on sharing protocols at https://plos.org/protocols?utm_medium=editorial-email&utm_source=authorletters&utm_campaign=protocols

# Author comment: Author response to Decision Letter 0

**DOI:** 10.1371/journal.pcbi.1008986.r002

29 Jul 2021

Attachment Submitted filename: PaIRKAT_Rebuttal.docx Click here for additional data file.

# Peer review: Decision Letter 1

**DOI:** 10.1371/journal.pcbi.1008986.r003

**Authors:** Mark Alber, Carl Herrmann

28 Aug 2021

Dear Carpenter,

Thank you very much for submitting your manuscript "PaIRKAT: A pathway integrated regression-based kernel association test with applications to metabolomics and COPD phenotypes" for consideration at PLOS Computational Biology.

As with all papers reviewed by the journal, your manuscript was reviewed by members of the editorial board and by several independent reviewers. In light of the reviews (below this email), we would like to invite the resubmission of a significantly-revised version that takes into account the reviewers' comments.

While Reviewer 1 is satisfied with the revisions to his/her comments, Reviewer 2 still feels that there is a lack of justification as to why the method is working. He/she feels that that additions made to the methods section are not sufficient to clearly illustrate this. As this is at the heart of your method, I would recommend that you add more explanations (1) in your response to the reviewer, and (2) add some additional explanations in the results/discussion, in addition to the methods.

We cannot make any decision about publication until we have seen the revised manuscript and your response to the reviewers' comments. Your revised manuscript is also likely to be sent to reviewers for further evaluation.

When you are ready to resubmit, please upload the following:

[1] A letter containing a detailed list of your responses to the review comments and a description of the changes you have made in the manuscript. Please note while forming your response, if your article is accepted, you may have the opportunity to make the peer review history publicly available. The record will include editor decision letters (with reviews) and your responses to reviewer comments. If eligible, we will contact you to opt in or out.

[2] Two versions of the revised manuscript: one with either highlights or tracked changes denoting where the text has been changed; the other a clean version (uploaded as the manuscript file).

Important additional instructions are given below your reviewer comments.

Please prepare and submit your revised manuscript within 60 days. If you anticipate any delay, please let us know the expected resubmission date by replying to this email. Please note that revised manuscripts received after the 60-day due date may require evaluation and peer review similar to newly submitted manuscripts.

Thank you again for your submission. We hope that our editorial process has been constructive so far, and we welcome your feedback at any time. Please don't hesitate to contact us if you have any questions or comments.

Sincerely,

Carl Herrmann, Ph.D.

Associate Editor

PLOS Computational Biology

Mark Alber

Deputy Editor

PLOS Computational Biology

***********************

Reviewer's Responses to Questions

**Comments to the Authors:**

**Please note here if the review is uploaded as an attachment.**

Reviewer #1: I appreciate the authors' responses to my previous comments.

Reviewer #2: The authors have addressed several of my concerns and I’m glad that they were able to change the p-value calculation approach.

However, I remain unclear as to the justification for the proposed approach. The “additions” made are not responsive to my question (with similar concern raised by other reviews). I still fail to understand *why* their approach works. The authors need to make a more solid conjecture with evidence to support their conjecture, by way of simulations or proof.

**********

**Have the authors made all data and (if applicable) computational code underlying the findings in their manuscript fully available?**

The PLOS Data policy requires authors to make all data and code underlying the findings described in their manuscript fully available without restriction, with rare exception (please refer to the Data Availability Statement in the manuscript PDF file). The data and code should be provided as part of the manuscript or its supporting information, or deposited to a public repository. For example, in addition to summary statistics, the data points behind means, medians and variance measures should be available. If there are restrictions on publicly sharing data or code —e.g. participant privacy or use of data from a third party—those must be specified.

Reviewer #1: None

Reviewer #2: Yes

**********

PLOS authors have the option to publish the peer review history of their article (what does this mean?). If published, this will include your full peer review and any attached files.

If you choose “no”, your identity will remain anonymous but your review may still be made public.

**Do you want your identity to be public for this peer review?** For information about this choice, including consent withdrawal, please see our Privacy Policy.

Reviewer #1: No

Reviewer #2: No

Figure Files:

While revising your submission, please upload your figure files to the Preflight Analysis and Conversion Engine (PACE) digital diagnostic tool, https://pacev2.apexcovantage.com. PACE helps ensure that figures meet PLOS requirements. To use PACE, you must first register as a user. Then, login and navigate to the UPLOAD tab, where you will find detailed instructions on how to use the tool. If you encounter any issues or have any questions when using PACE, please email us at figures@plos.org.

Data Requirements:

Please note that, as a condition of publication, PLOS' data policy requires that you make available all data used to draw the conclusions outlined in your manuscript. Data must be deposited in an appropriate repository, included within the body of the manuscript, or uploaded as supporting information. This includes all numerical values that were used to generate graphs, histograms etc.. For an example in PLOS Biology see here: http://www.plosbiology.org/article/info%3Adoi%2F10.1371%2Fjournal.pbio.1001908#s5.

Reproducibility:

To enhance the reproducibility of your results, we recommend that you deposit your laboratory protocols in protocols.io, where a protocol can be assigned its own identifier (DOI) such that it can be cited independently in the future. Additionally, PLOS ONE offers an option to publish peer-reviewed clinical study protocols. Read more information on sharing protocols at https://plos.org/protocols?utm_medium=editorial-email&utm_source=authorletters&utm_campaign=protocols

# Author comment: Author response to Decision Letter 1

**DOI:** 10.1371/journal.pcbi.1008986.r004

11 Oct 2021

Attachment Submitted filename: PaIRKAT_Re_Rebuttal.docx Click here for additional data file.

# Peer review: Decision Letter 2

**DOI:** 10.1371/journal.pcbi.1008986.r005

**Authors:** Mark Alber, Carl Herrmann

13 Oct 2021

Dear Carpenter,

We are pleased to inform you that your manuscript 'PaIRKAT: A pathway integrated regression-based kernel association test with applications to metabolomics and COPD phenotypes' has been provisionally accepted for publication in PLOS Computational Biology.

Before your manuscript can be formally accepted you will need to complete some formatting changes, which you will receive in a follow up email. A member of our team will be in touch with a set of requests.

Please note that your manuscript will not be scheduled for publication until you have made the required changes, so a swift response is appreciated.

IMPORTANT: The editorial review process is now complete. PLOS will only permit corrections to spelling, formatting or significant scientific errors from this point onwards. Requests for major changes, or any which affect the scientific understanding of your work, will cause delays to the publication date of your manuscript.

Should you, your institution's press office or the journal office choose to press release your paper, you will automatically be opted out of early publication. We ask that you notify us now if you or your institution is planning to press release the article. All press must be co-ordinated with PLOS.

Thank you again for supporting Open Access publishing; we are looking forward to publishing your work in PLOS Computational Biology. 

Best regards,

Carl Herrmann, Ph.D.

Associate Editor

PLOS Computational Biology

Mark Alber

Deputy Editor

PLOS Computational Biology

***********************************************************

Reviewer's Responses to Questions

**Comments to the Authors:**

**Please note here if the review is uploaded as an attachment.**

Reviewer #2: The authors have sufficiently addressed my concerns.

**********

**Have the authors made all data and (if applicable) computational code underlying the findings in their manuscript fully available?**

The PLOS Data policy requires authors to make all data and code underlying the findings described in their manuscript fully available without restriction, with rare exception (please refer to the Data Availability Statement in the manuscript PDF file). The data and code should be provided as part of the manuscript or its supporting information, or deposited to a public repository. For example, in addition to summary statistics, the data points behind means, medians and variance measures should be available. If there are restrictions on publicly sharing data or code —e.g. participant privacy or use of data from a third party—those must be specified.

Reviewer #2: Yes

**********

PLOS authors have the option to publish the peer review history of their article (what does this mean?). If published, this will include your full peer review and any attached files.

If you choose “no”, your identity will remain anonymous but your review may still be made public.

**Do you want your identity to be public for this peer review?** For information about this choice, including consent withdrawal, please see our Privacy Policy.

Reviewer #2: No

# Editor report: Acceptance letter

**DOI:** 10.1371/journal.pcbi.1008986.r006

**Authors:** Mark Alber, Carl Herrmann

15 Oct 2021

PCOMPBIOL-D-21-00744R2 

PaIRKAT: A pathway integrated regression-based kernel association test with applications to metabolomics and COPD phenotypes

Dear Dr Carpenter,

I am pleased to inform you that your manuscript has been formally accepted for publication in PLOS Computational Biology. Your manuscript is now with our production department and you will be notified of the publication date in due course.

The corresponding author will soon be receiving a typeset proof for review, to ensure errors have not been introduced during production. Please review the PDF proof of your manuscript carefully, as this is the last chance to correct any errors. Please note that major changes, or those which affect the scientific understanding of the work, will likely cause delays to the publication date of your manuscript. 

Soon after your final files are uploaded, unless you have opted out, the early version of your manuscript will be published online. The date of the early version will be your article's publication date. The final article will be published to the same URL, and all versions of the paper will be accessible to readers.

Thank you again for supporting PLOS Computational Biology and open-access publishing. We are looking forward to publishing your work! 

With kind regards,

Livia Horvath

PLOS Computational Biology | Carlyle House, Carlyle Road, Cambridge CB4 3DN | United Kingdom ploscompbiol@plos.org | Phone +44 (0) 1223-442824 | ploscompbiol.org | @PLOSCompBiol
